# Intelligence-enabled smart inhalable biomaterials for chronic inflammatory airway diseases: integrating responsive carrier engineering, lung-on-chip validation, and patient-specific pulmonary modeling

**DOI:** 10.3389/fbioe.2026.1964825

**Published:** 2026-10-15

**Authors:** Qi Fan, Zhongxu Li, Yongliang Sun, Yu Liu, Hangyu Chen

**Affiliations:** 1 Changchun University of Chinese Medicine, Changchun, China; 2 The Affiliated Hospital to Changchun University of Chinese Medicine, Changchun, China; 3 Linjiang Hospital of Traditional Chinese Medicine, Jilin, China; 4 The Third Affiliated Hospital of Changchun University of Chinese Medicine, Changchun, China

**Keywords:** asthma, chronic obstructive pulmonary disease, computational fluid dynamics, lung-on-chip, machine learning, mucus penetration, pulmonary drug delivery, smart inhalable biomaterials

## Abstract

Chronic inflammatory airway diseases, particularly asthma and chronic obstructive pulmonary disease (COPD), are commonly treated by inhalation, yet the nominal dose placed in an inhaler often differs substantially from the dose that reaches diseased airway regions, crosses mucus, enters target cells, and remains pharmacologically active. Airway narrowing, heterogeneous ventilation, mucus plugging, impaired mucociliary clearance, oxidative and proteolytic stress, epithelial barrier dysfunction, inflammatory-cell diversity, device characteristics, and patient-specific inhalation maneuvers jointly shape therapeutic exposure. Smart inhalable biomaterials can be designed to address some of these barriers by controlling transport, retention, activation, or release, but many systems are still optimized primarily at the nanoparticle level and evaluated in static models that do not capture realistic aerosol performance or patient-specific deposition. This review develops an intelligence-enabled bioengineering framework linking disease-informed material design with machine-learning-assisted formulation optimization, formulation–device–patient coupling, lung-on-chip validation, and patient-specific pulmonary modeling. Within this framework, airway features such as mucus architecture, redox gradients, protease activity, epithelial permeability, immune-cell state, extracellular-matrix remodeling, and regional airflow are considered measurable variables that can inform material design and evaluation. The material platforms considered include mucus-penetrating and mucoadhesive carriers, responsive materials, transformable nano-in-micro systems, polymeric and lipid nanoparticles, biomimetic carriers, extracellular vesicles, and systems for pulmonary delivery of nucleic acids and biologics. Their development is considered alongside structured experimental design, machine learning, active learning, mechanistic aerosol modeling, physics-informed surrogates, multi-objective optimization, and uncertainty analysis. Lung-on-chip systems provide human-relevant settings in which deposited dose, mucus transport, barrier function, immune responses, and repeated-exposure effects can be examined under air–liquid-interface and breathing-like conditions. Patient-specific computational fluid dynamics and reduced-order models can complement these platforms by predicting regional deposition, whereas the term digital twin should be reserved for longitudinally updated, uncertainty-aware models linked to actionable decisions. Together, these approaches support a closed-loop design–delivery–validation strategy in which material performance, aerosol behavior, biological response, and patient-specific deposition are evaluated as connected rather than separate development problems.

## 1 Introduction

Inhalation is a rational route for chronic inflammatory airway diseases because it can achieve high local drug concentrations while limiting systemic exposure (Dolovich and Dhand, 2011). The apparent simplicity of this principle conceals a multiscale delivery problem. Between device actuation and therapeutic action, a formulation must be emitted reproducibly, form an appropriate aerosol, traverse the oral cavity and pharynx, enter airways characterized by heterogeneous airflow and obstruction, deposit in the desired region, avoid rapid clearance, cross or interact with mucus, remain chemically and structurally intact, and reach the relevant epithelial, immune, stromal, or airway smooth-muscle cells. Failure at any step can substantially diminish the value of a sophisticated drug or carrier. Accordingly, inhaled therapy is better understood as a coupled system consisting of a formulation, a delivery device, a patient-specific inhalation maneuver, an anatomically heterogeneous lung, and a dynamic disease microenvironment.

The gap between nominal dose and regional pulmonary exposure is particularly relevant in asthma and COPD. In asthma, variable bronchoconstriction, airway hyperresponsiveness, mucus plugging, epithelial alarmin release, type 2 inflammation, neutrophilic inflammation, and airway remodeling coexist in different proportions. In COPD, emphysema, small-airway fibrosis, chronic bronchitis, mucus hypersecretion, bacterial colonization, oxidative injury, macrophage dysfunction, and epithelial senescence create similarly heterogeneous delivery conditions. These disease processes are not distributed uniformly. The same patient may have relatively patent central airways, severely narrowed small airways, mucus-obstructed segments, and poorly ventilated regions. A formulation optimized for an average airway geometry or based on a single inflammatory marker may therefore perform well in one compartment but fail in another (De Backer et al., 2013).

In this review, “smart inhalable biomaterials” are defined by functional behavior rather than by material class. A polymeric nanoparticle, lipid nanoparticle, inorganic carrier, biomimetic particle, or extracellular vesicle is therefore not considered smart solely because of its composition, nanoscale structure, or use for pulmonary delivery. We use the term for carriers that incorporate at least one engineered function that is condition-dependent or context-selective and is mechanistically linked to a measurable delivery or therapeutic advantage. Qualifying functions include stimulus-responsive activation or release, programmed transformation of size, charge, surface exposure, or structure, disease-microenvironment-dependent behavior, and demonstrable cell- or compartment-selective interactions. Fixed surface modification alone—for example, constitutive PEGylation, size optimization, or addition of a ligand—is not considered sufficient unless it produces a demonstrated context-selective function or forms part of a programmed responsive or sequential delivery mechanism.

Smart inhalable biomaterials seek to convert this heterogeneity into a therapeutic opportunity (Mansour et al., 2024). A surface can be engineered to minimize adhesive interactions with mucins; a coating can detach after encountering an enzyme; a linker can cleave under oxidative or reducing conditions; a particle can transform after aerosol deposition; or a carrier can be preferentially internalized by macrophages or epithelial cells. These strategies are often described as targeted or responsive. Yet responsiveness alone does not ensure therapeutic relevance. A carrier may respond only at a stimulus concentration not reached in human airway lining fluid, may release before reaching the intended site, may lose activity during spray drying or nebulization, or may be too cohesive to aerosolize efficiently. A central design question is therefore whether the material response addresses a quantitatively demonstrated barrier under realistic delivery conditions.

A second limitation is fragmentation across disciplines. Material scientists commonly optimize hydrodynamic size, zeta potential, encapsulation efficiency, and release in bulk solution. Aerosol scientists focus on emitted dose, mass median aerodynamic diameter (MMAD), geometric standard deviation (GSD), and fine particle fraction (FPF). Cell biologists evaluate inflammatory and barrier responses, whereas computational groups estimate regional flow and deposition. These measurements are valuable but are not consistently connected within an iterative decision framework. As a result, a carrier that appears optimal at the nanoscale may aerosolize poorly; a nano-in-micro powder with excellent FPF may release nanoparticles that become trapped in disease-relevant mucus; and a favorable animal response observed after intratracheal instillation may not translate to clinically relevant inhalation (Bahadori and Mohammadi, 2012).

The proposed intelligence-enabled framework differs from the conventional inhaled drug-development workflow primarily in how decisions are organized, rather than simply in the addition of computational tools. Conventional development is often sequential: a carrier is formulated and optimized for physicochemical properties, aerosol performance is subsequently evaluated with a selected device, biological activity is tested in relatively standardized models, and patient-specific delivery variability is considered mainly at later translational stages. In the framework proposed here, development instead begins with a defined disease-specific delivery bottleneck that is translated into quantitative engineering specifications. Material, formulation, device, biological, and deposition variables are then evaluated as coupled design dimensions, while experimental and computational outputs are iteratively returned to guide subsequent candidate selection. Lung-on-chip systems and patient-specific pulmonary models therefore function not as downstream validation add-ons, but as sources of decision-relevant feedback within the development cycle. The fundamental distinction is thus between a predominantly sequential, component-wise workflow and an iterative, feedback-driven workflow in which decisions at each stage are informed by measurements generated across the delivery system.

The term intelligence-enabled is used here in a restrictive operational sense. A development workflow is not considered intelligence-enabled merely because it incorporates statistical design, molecular simulation, computational fluid dynamics, image analysis, machine learning, or uncertainty quantification. We reserve the term for iterative decision-making workflows in which (i) structured experimental or simulation data are used to construct or update a decision model; (ii) the model prospectively prioritizes candidate formulations, experiments, device conditions, or other actionable design choices; (iii) the selected candidates are evaluated experimentally or through predefined higher-fidelity validation; and (iv) the resulting observations are returned to update the model and inform a subsequent round of decision-making. Conventional computational modeling can provide mechanistic or predictive insight without such feedback, whereas data-driven optimization can rank or prioritize candidates within a fixed dataset without necessarily updating the model prospectively. Closed-loop intelligence-enabled development therefore requires prospective model-guided selection, validation, feedback, and model updating. Methods such as design of experiments, mechanistic modeling, image analysis, reduced-order modeling, and uncertainty quantification can support this loop but are not, by themselves, sufficient to qualify a workflow as intelligence-enabled (Boso et al., 2020).

Accordingly, we distinguish three levels of computational involvement throughout this review. Conventional computational or statistical tools analyze, simulate, or structure a predefined problem but do not themselves determine subsequent experimental actions. Data-driven decision-support approaches use learned relationships or surrogate models to predict, rank, or optimize candidates, but may still operate on a fixed dataset. Fully closed-loop intelligence-enabled development begins when model outputs prospectively determine what is tested next and the resulting data are fed back to update the model and subsequent decisions. Many currently reported studies in inhalable biomaterials represent enabling components or intermediate stages of this continuum rather than fully realized closed-loop systems.

This review focuses on asthma and COPD as representative chronic inflammatory airway diseases and develops a closed-loop pathway: disease features are translated into engineering specifications; carrier and device variables are co-optimized; emitted formulations are evaluated in human-relevant lung-on-chip systems; patient-specific pulmonary models estimate regional deposition; and the resulting data are returned to the design stage. Figure 1 summarizes how disease-informed material design, formulation–device optimization, human-relevant validation, and patient-specific pulmonary modeling are connected within this framework. The emphasis is on bioengineering rather than clinical risk prediction.

**FIGURE 1:**
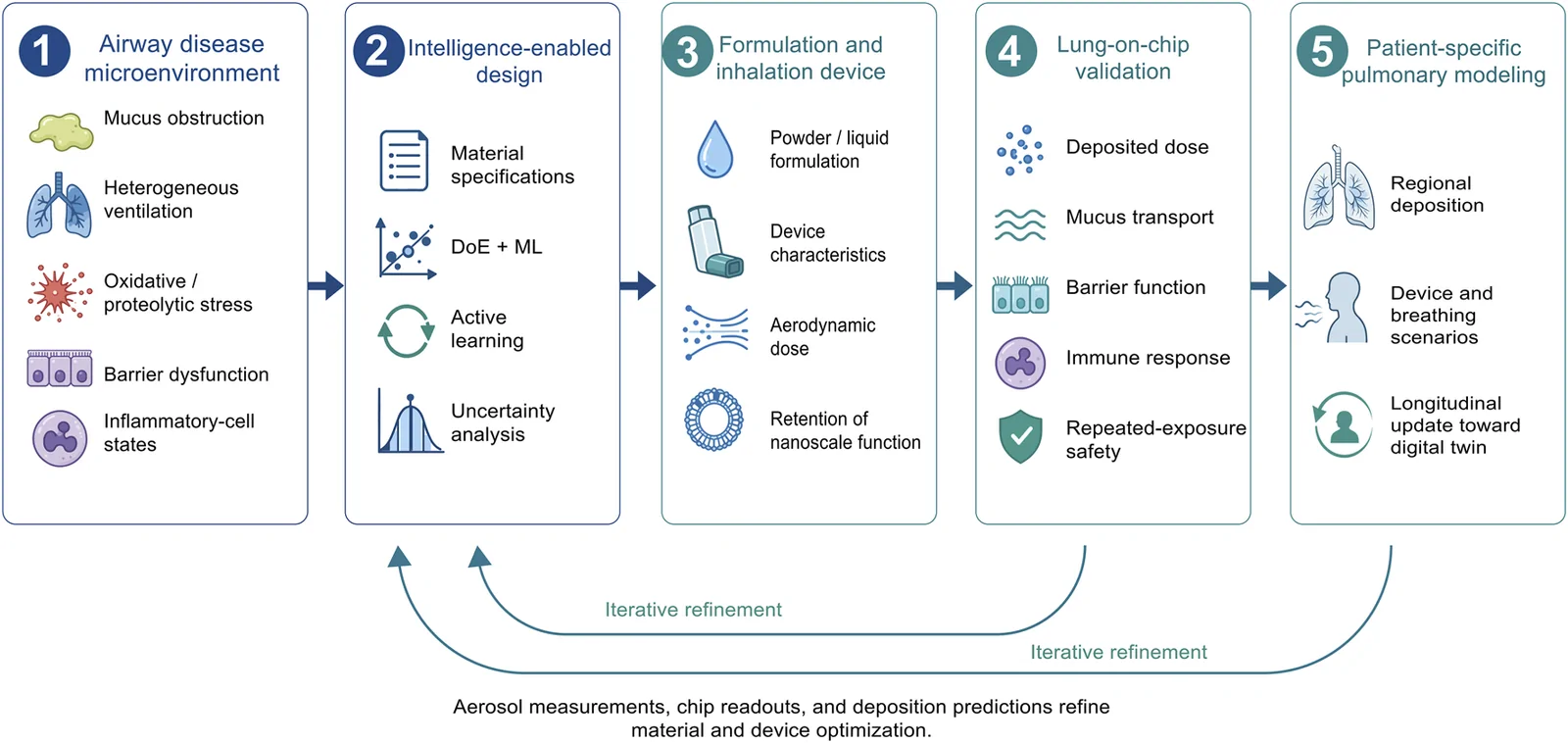
Closed-loop bioengineering framework for intelligence-enabled smart inhalable biomaterials. Chronic inflammatory airway features, including mucus obstruction, heterogeneous ventilation, oxidative and proteolytic stress, epithelial barrier dysfunction, extracellular-matrix remodeling, and inflammatory-cell states, are translated into quantitative material specifications. Carrier composition, powder or liquid formulation, manufacturing process, device characteristics, and inhalation waveform are optimized jointly using design of experiments, machine learning, active learning, mechanistic aerosol models, and uncertainty analysis. Candidate formulations are emitted through the intended inhalation device and characterized for dose, aerodynamic distribution, and retention of nanoscale function. Dynamic airway or breathing lung chips then measure deposited dose, mucus transport, epithelial and endothelial barrier function, immune-cell response, target engagement, and repeated-exposure safety. In parallel, patient-specific or representative disease geometries are used to estimate regional deposition under alternative devices and breathing maneuvers. Aerosol measurements, chip readouts, and deposition predictions are returned to the design stage for iterative refinement. A true pulmonary digital twin represents a later stage in which imaging, lung function, inhaler-use data, biomarkers, and treatment response update the model longitudinally; uncertainty is quantified; and model output is linked to a validated formulation, device, dose, or clinical decision.

## 2 Chronic airway disease as a multiscale delivery problem

### 2.1 Asthma: endotypes, mucus obstruction, and variable target biology

Asthma is not a single inflammatory state. Type 2-high disease may be characterized by interleukin (IL)-4, IL-5, IL-13, eosinophils, immunoglobulin E, epithelial alarmins, and goblet-cell metaplasia, whereas type 2-low disease may involve neutrophilic or paucigranulocytic inflammation, altered innate immune responses, obesity-associated mechanisms, infection-associated inflammation, or corticosteroid insensitivity (Woodruff et al., 2009). These inflammatory endotypes and phenotypes may alter the identity and abundance of target cells, the composition and rheology of mucus, the concentrations of extracellular enzymes and oxidants, and the likely response to a delivered cargo. A carrier intended to deliver a nucleic acid to epithelial cells, for example, generally requires efficient mucus penetration and cellular uptake, whereas an extracellular antiprotease or mucolytic intervention may benefit from controlled residence within the mucus layer.

Mucus plugs have emerged as a structurally and clinically important feature of severe asthma. Plugs can persist in the same airway segments, contribute to ventilation defects, and are associated with airflow obstruction and type 2 inflammation (Tamura et al., 2022; Tran et al., 2022; Tanabe et al., 2025; Aegerter et al., 2026). From a materials perspective, a plug is not simply a thicker version of healthy mucus. It may contain concentrated mucins, extracellular DNA, actin, eosinophil-derived products, cellular debris, altered ionic composition, and heterogeneous hydration. Diffusion measured in dilute commercial mucin therefore cannot be assumed to predict transport through disease-derived sputum or an intact plug. Spatial variability also means that a single surface chemistry may encounter substantially different barriers along the respiratory tree.

Spatial heterogeneity of mucus plugging also has direct aerodynamic consequences. Segmental luminal narrowing or occlusion can redistribute airflow toward more patent airway regions, alter local flow velocity, and increase the likelihood of proximal or focal deposition while reducing aerosol penetration downstream of obstructed segments. Consequently, a single nominal aerodynamic diameter may not provide uniform exposure across a lung containing relatively open, narrowed, and mucus-obstructed airways. Smaller or extrafine respirable particles may improve access to distal and small airways relative to larger particles, but reduced particle size alone cannot fully compensate for severe or spatially heterogeneous obstruction. Recent patient-specific CFD analyses in severe asthma showed that airway morphology and mucus burden were associated with regional inhaled corticosteroid deposition; extrafine particles achieved greater central and peripheral deposition than fine particles, but did not completely overcome morphology-associated underdelivery (Ragunayakam et al., 2025). Aerodynamic design for mucus-plugged asthma should therefore consider not only MMAD, but also particle-size distribution, particle density and hygroscopic behavior, device-generated aerosol characteristics, and the inhalation waveform in relation to the spatial distribution of airway obstruction.

Airway remodeling adds a temporal dimension. Repeated epithelial injury and inflammation can promote reticular basement-membrane thickening, increased airway smooth-muscle mass, fibroblast activation, extracellular-matrix deposition, and altered innervation (Kaminska et al., 2009; Drake et al., 2018). These changes affect airway caliber and mechanics while also modifying the therapeutic target. A material designed only to suppress acute inflammatory signaling may not address persistent epithelial dysfunction or matrix remodeling. Conversely, an anti-remodeling intervention intended for sustained action may require sustained local exposure, careful control of systemic exposure, and repeated-dose safety over clinically relevant treatment periods.

### 2.2 COPD: small-airway disease, oxidative stress, and macrophage dysfunction

COPD combines persistent airflow obstruction with variable proportions of emphysema, small-airway remodeling, chronic bronchitis, mucus hypersecretion, inflammation, airway infection or bacterial colonization, and systemic comorbidities. Small conducting airways are a major site of airflow obstruction and remodeling, but they are also difficult to access by inhaled particles when upstream narrowing and heterogeneous ventilation reduce distal delivery and favor more proximal deposition (Hogg et al., 2004). Emphysematous regions may exhibit marked ventilation heterogeneity together with altered surface area, perfusion, and mechanical behavior. Chronic bronchitic regions can be mucus-rich and poorly cleared. A formulation intended for COPD therefore requires not only a respirable aerodynamic profile but also an explicit target compartment.

Oxidative stress is a major but heterogeneous pathogenic feature of COPD. Exogenous oxidants from cigarette smoke or air pollution are compounded by endogenous reactive oxygen species production by activated neutrophils, macrophages, and epithelial cells (Barnes, 2020). Oxidative burden can inactivate antiproteases, alter mucus, damage mitochondria, activate nuclear factor-kappa B and inflammasome pathways, and promote cellular senescence. These mechanisms motivate antioxidant and redox-responsive materials, yet they also create design ambiguity. During airway inflammation, the extracellular airway environment may exhibit increased oxidative burden, whereas the cytosol of a target cell generally remains comparatively reducing because it contains millimolar concentrations of glutathione. A carrier may exploit this sequence by remaining stable during aerosolization and mucus transit, then releasing cargo after cellular internalization through disulfide cleavage. A recent inhalable nanotherapeutic system for COPD used glutathione-responsive disassembly to support macrophage reprogramming (Oh et al., 2026). Such systems illustrate the value of distinguishing extracellular and intracellular redox compartments rather than treating “ROS responsiveness” as a single phenomenon.

Alveolar and airway macrophages are attractive targets because inhaled particles are readily taken up by pulmonary phagocytic cells (Akata and van Eeden, 2020). This uptake can be advantageous for therapies intended to modulate macrophage inflammatory states, restore efferocytosis, suppress inflammasome signaling, or deliver antimicrobial agents. The same uptake can also be a liability: rapid sequestration may prevent epithelial delivery, persistent inorganic material may accumulate, and a cationic or highly reactive surface may intensify inflammation. Macrophage targeting should therefore be assessed in the context of cell state, dose, biodegradation, and repeated exposure. A higher uptake percentage is not automatically beneficial if it reflects membrane injury or nonspecific particle sequestration rather than productive intracellular delivery.

### 2.3 Exacerbations and temporal heterogeneity

Both asthma and COPD fluctuate between relatively stable periods and exacerbations driven by viral infection, bacterial infection, pollution, allergen exposure particularly in asthma, nonadherence, or treatment interruption (Jackson et al., 2011). The airway microenvironment during an exacerbation may differ markedly from the stable state: depending on the disease and trigger, mucus burden and viscoelasticity may increase, pH and ionic conditions may change, protease and oxidant activity may rise, epithelial barrier dysfunction may worsen, and inspiratory performance may deteriorate. A patient who can generate an adequate inspiratory flow through a dry powder inhaler when stable may generate insufficient device-specific inspiratory flow during a severe exacerbation (Sharma et al., 2017). Thus, the same material–device combination may not be optimal across disease states.

Temporal heterogeneity creates two potential strategies. One is robust design: a formulation is engineered to maintain acceptable aerosol and biological performance across a wide range of mucus properties, airflow conditions, and inflammatory states. The other is adaptive design: device selection, dose, or formulation strategy is changed according to the measured disease state. Adaptive therapy is conceptually aligned with digital twins and connected inhalers, but it requires reliable sensing, timely model updating, and evidence that adaptation improves decision quality or clinically meaningful outcomes (Gonsard et al., 2024). Until such infrastructure exists, preclinical development should at least test candidate systems across stable-like and exacerbation-like conditions rather than within a single standardized environment.

## 3 Inhalation engineering fundamentals that constrain smart materials

### 3.1 Aerodynamic diameter and regional deposition

Hydrodynamic diameter characterizes the effective size of a particle dispersed in liquid, whereas aerodynamic diameter determines how an aerosolized particle behaves in air. Aerodynamic behavior depends on geometric diameter, density, shape, and the aerodynamic shape factor. This distinction is central to inhaled nanomedicine. Many particles in the submicron range have limited deposition by inertial impaction or sedimentation and may be exhaled, although sufficiently small ultrafine particles can deposit through diffusion; larger or denser agglomerates may instead impact in the mouth or central airways. Nano-in-micro strategies address this challenge by incorporating nanoparticles into respirable microparticles that dissolve or disintegrate after deposition to release the nanocarriers (Baghdan et al., 2019). The success of such systems depends on two linked events: the microparticle must aerosolize and deposit appropriately, and the released nanocarrier must retain its intended size, surface properties, and cargo integrity.

Impaction dominates for larger particles and high-velocity flows, sedimentation becomes important in low-flow peripheral airways and during breath holding, and diffusion contributes increasingly to the deposition of ultrafine particles. Hygroscopic growth, evaporation, electrostatic charge, agglomeration, and airway geometry can alter the effective aerodynamic size and regional deposition pattern. A single MMAD value does not capture the full distribution, nor does a high FPF guarantee delivery to a specific diseased region. Regional deposition should be interpreted together with device plume characteristics, inhalation waveform, airway obstruction, and the target site in mind (Bartol et al., 2024; Sadafi et al., 2024a; Sadafi et al., 2024b).

For chronic airway disease, the desired deposition region is not always the peripheral or alveolar lung. Bronchodilators and anti-inflammatory agents may need broad conducting-airway coverage, depending on their intended site of action, whereas therapies targeting alveolar macrophages or alveolar epithelial cells generally require delivery to distal or alveolar regions. Small-airway targeting may benefit from appropriately small respirable particles and a device-appropriate inhalation maneuver, but severe obstruction can reduce particle penetration into downstream regions (Usmani et al., 2005). A rational development program should therefore define a target compartment before optimizing aerosol metrics.

The aerodynamic particle-size distribution measured after device actuation is also device dependent and should not be assumed to reflect the pre-device formulation alone. In dry powder inhalers, patient-generated pressure drop, device resistance, internal turbulence, and particle–particle or particle–device interactions govern powder deagglomeration and therefore influence the size distribution and fine-particle fraction of the emitted aerosol. In contrast, soft-mist inhalers generate aerosols from liquid formulations through mechanically driven atomization, and the resulting droplet-size distribution depends on nozzle design and operating conditions as well as formulation properties such as viscosity and surface tension (Komalla et al., 2023). Passage through the device may also alter the integrity of complex carriers; for example, atomization-associated stresses can promote aggregation or cargo leakage from lipid nanoparticles, depending on the formulation and aerosol-generation method (Miao et al., 2023). Aerodynamic characterization should therefore be performed on the aerosol actually emitted from the intended device, together with post-device assessment of carrier size, integrity, and biological function, rather than inferred solely from hydrodynamic measurements of the formulation before aerosolization.

### 3.2 Formulation-device-patient coupling

Pressurized metered-dose inhalers, dry powder inhalers, soft-mist inhalers, jet nebulizers, ultrasonic nebulizers, and vibrating-mesh nebulizers impose different physical stresses and patient requirements. Dry powder performance depends on powder dispersibility and the pressure drop and flow profile generated by the patient through the device (Jõgi et al., 2021). Pressurized metered-dose inhalers require coordination unless used with a spacer or a breath-actuated mechanism. Nebulizers reduce the need for high inspiratory flow but, depending on their design, may expose a formulation to shear, air–liquid interfaces, recirculation, heating or concentration changes, and prolonged residence in the device (Lehofer et al., 2014). Smart materials must therefore be developed with the intended device in mind rather than transferred between platforms late in development.

Device compatibility extends beyond emitted dose. Nebulization may alter particle size, disrupt liposomes, compromise protein structure or activity, release cargo prematurely, or promote foaming (Lehofer et al., 2014). Spray drying can expose biomolecules to thermal, interfacial, and dehydration stresses, while freeze drying can produce aggregation without suitable cryoprotectants or lyoprotectants. Excipients that improve powder dispersibility may alter cellular uptake or mucus interactions. A responsive linkage that performs well in a vial may undergo premature hydrolysis or other degradation during storage or processing. These possibilities justify an early stress-testing sequence that includes manufacturing, storage, device passage, aerosol collection, reconstitution where relevant, and assessment of post-aerosol physicochemical integrity and biological function.

The patient is an active component of the system. Inspiratory flow, inhaled volume, flow acceleration, breath-hold duration, disease severity, age, cognition, and technique influence delivery. Device resistance shapes the pressure drop, flow profile, and powder deaggregation, and its suitability depends on the patient’s ability to generate sufficient inspiratory effort and volume. Connected inhalers can document actuation and some aspects of inhalation behavior; these data are useful for assessing adherence and technique, and their value for delivery optimization increases when they are linked to deposition or exposure estimates (Levy et al., 2024). More broadly, IoT-based health-monitoring frameworks coupled with attention-based deep-learning approaches illustrate how continuously acquired sensor data can be integrated for automated health-state assessment (Nainwal et al., 2025). In an intelligence-enabled workflow, patient-use data can inform model inputs and device selection rather than being treated solely as adherence metrics.

### 3.3 Dose nomenclature and pharmacokinetic fate

Device-loaded dose, nominal dose, delivered (emitted) dose, fine-particle dose, lung-deposited dose, retained dose, tissue-exposed dose, internalized dose, and pharmacologically active dose describe different stages of delivery and should not be treated as interchangeable (Wolff, 2021). Many preclinical studies report only the amount placed in an administration device or administered by intratracheal instillation. This makes comparisons difficult and can overstate apparent pulmonary delivery efficiency or translational relevance (Ehrmann et al., 2020). For smart inhalable biomaterials, dose accounting should separately track the carrier and cargo from formulation loading through device emission, aerodynamic size distribution, regional deposition, clearance or retention, cellular uptake, cargo release, and elimination.

After deposition, a carrier may be transported by mucociliary clearance and subsequently swallowed, phagocytosed, dissolved, degraded, translocated across the epithelium, or, in some cases, redistributed through draining lymphatics or the systemic circulation (Labiris and Dolovich, 2003). Once dissolved, permeable small molecules may be absorbed rapidly into the pulmonary circulation, whereas poorly soluble, slowly degradable, or strongly retained systems may persist locally and create cumulative exposure. Biodegradability should therefore not be treated as sufficient evidence of pulmonary safety; the identities, formation and clearance kinetics, and toxicity of degradation products should be characterized. Repeated-dose studies are particularly important for chronic disease because a material that is well tolerated after a single exposure may accumulate or alter macrophage function after repeated administration.

### 3.4 Aerosol-to-chip dosimetry, transmission efficiency, and mass balance

Aerosol-to-tissue dosimetry should be treated as a distinct engineering problem rather than inferred from the amount initially loaded into an inhalation device. Device-loaded dose, emitted aerosol, aerosol entering an exposure chamber or chip, and material ultimately deposited on the tissue represent different stages of the delivery pathway and may differ substantially because of retention and losses within the device and exposure system. For *in vitro* aerosol exposure, quantitative interpretation therefore requires a mass-balance framework that follows the formulation from device emission to tissue deposition.

A useful first parameter is aerosol transmission efficiency, defined operationally as the fraction of emitted aerosol that reaches the exposure chamber or chip inlet. Transmission can be affected by tubing length and diameter, bends and connectors, electrostatic deposition, gravitational settling, inertial impaction, condensation, and interactions with chamber surfaces. These losses may also be size dependent, such that the particle or droplet distribution reaching the tissue differs from that measured immediately downstream of the inhalation device. Consequently, aerosol size distribution should, where feasible, be characterized at or near the exposure-system inlet rather than assumed to remain unchanged during transport.

Deposition efficiency and spatial uniformity should then be considered separately from transmission. A high aerosol concentration within an exposure chamber does not necessarily imply a high or spatially uniform tissue-deposited dose. Local flow patterns, chamber geometry, impaction, sedimentation, and diffusion can generate substantial differences among chip positions or across the exposed tissue surface. Quartz crystal microbalances, fluorescence-based measurements, chemical assays, radiolabeling, gravimetric approaches, or collected-mass measurements can be used, depending on the formulation and analyte, to quantify deposited mass (Ding et al., 2020). When multiple chips or wells are exposed simultaneously, spatial mapping of deposition is important to distinguish biological variability from dosimetric heterogeneity.

Evaporation introduces an additional source of uncertainty for liquid aerosols. During transport from the nebulizer to the tissue surface, water loss can reduce droplet size while increasing the local concentration of drug, salts, excipients, or nanoparticles within the remaining droplet. The aerosol arriving at the tissue may therefore differ in both aerodynamic behavior and formulation composition from the initially generated aerosol. Temperature, relative humidity, residence time, airflow, and exposure-system geometry should consequently be controlled or reported when evaporation is likely to influence exposure.

Where technically feasible, overall mass recovery should be reported to determine whether the major fractions of the administered aerosol have been accounted for. Depending on the experimental configuration, this may include residual material in the inhalation device, material retained within connectors or tubing, deposits on chamber surfaces, tissue-deposited material, and material collected downstream or at the exhaust. The denominator used for recovery should be stated explicitly, because recovery relative to device-loaded dose and recovery relative to emitted dose address different questions. Incomplete recovery can indicate unrecognized deposition, leakage, sampling limitations, or transformation of the formulation during aerosol transport.

These measurements are particularly important for smart or biologically active carriers because aerosol transmission can alter more than total dose. Selective particle loss, evaporation, aggregation, cargo leakage, or disruption of responsive or biomimetic structures may change the physicochemical identity and biological potency of the material that actually reaches the tissue. Accordingly, aerosol-to-chip studies should ideally report emitted dose, transmission efficiency, aerosol characteristics at the exposure-system inlet, deposited dose, deposition uniformity, overall mass recovery, and post-aerosol carrier integrity. These parameters provide the quantitative link required to interpret biological responses in air–liquid-interface and lung-on-chip systems.

## 4 Translating airway microenvironments into quantitative material specifications

### 4.1 Mucus architecture, adhesion, and penetration

Airway mucus is a hydrated polymer network structured primarily by secreted mucins, while its properties are influenced by salts, proteins, lipids, DNA, actin, extracellular vesicles, and, particularly in disease, inflammatory cells and microbial products (Pangeni et al., 2023). In health, coordinated secretion, hydration, ciliary beating, and, when required, cough support clearance. In chronic inflammatory airway diseases, increased mucin production, dehydration, altered crosslinking, and accumulated macromolecules can increase viscoelasticity and impair mucus transport. Mucus is therefore both a biochemical and mechanical barrier.

Particle interaction with mucus is strongly influenced by particle size relative to the local mesh spacing and by adhesive interactions. Hydrophobic, electrostatic, and hydrogen-bonding interactions can immobilize particles even when they are smaller than the estimated local mesh spacing. Dense coatings of low-molecular-weight polyethylene glycol can reduce these interactions, allowing 100–200 nm particles to diffuse more rapidly in human respiratory mucus, while selected zwitterionic chemistries may provide a similarly mucus-inert surface (Schuster et al., 2013; Schneider et al., 2017). Mucus-penetrating nanoparticles have produced more uniform and sustained airway distribution than similarly sized mucoadhesive particles after inhalation (Schneider et al., 2017). The principle has also supported airway gene delivery in muco-obstructive models (Kim et al., 2022).

The same properties that enable mucus-penetrating particles to evade adhesive trapping and mucociliary removal also create potential safety trade-offs. Prolonged residence and closer access to the airway epithelium can increase epithelial exposure, while particles that reach more distal pulmonary regions may encounter slower clearance pathways and different macrophage or translocation mechanisms. These concerns are particularly relevant for poorly degradable materials or repeated administration, for which prolonged pulmonary retention, cellular accumulation, or redistribution beyond the airway lumen may become important. Mucus penetration should therefore not be optimized independently of biodegradability, clearance kinetics, epithelial compatibility, and repeated-dose safety. For chronic inhalation, the desired design is not maximal retention but sufficient residence to achieve target engagement followed by predictable degradation or clearance.

Mucus penetration and mucoadhesion should not be framed as universally beneficial or detrimental strategies. Mucoadhesion may increase local interaction or retention within mucus for a drug acting on mucus, pathogens, or extracellular enzymes, although the benefit depends on mucus turnover and mucociliary clearance. Penetration is preferable when the target lies beneath the mucus or when broader epithelial distribution is required. Hybrid and sequential strategies may help reconcile these competing requirements: a carrier can be mucus-inert during transit, then expose an adhesive or cell-interactive surface after reaching the epithelium. Charge-reversal systems, detachable shells, enzyme-cleavable coatings, and transformable particles are potential examples, although each added function complicates characterization.

Quantitative evaluation should include particle-tracking-derived diffusion coefficients, the distribution of individual-particle diffusivities rather than only an ensemble mean, mobile fraction, penetration depth, retention under controlled flow, and epithelial uptake after mucus traversal. Mucus source is critical. Reconstituted commercial mucin solutions or gels lack the structural complexity of native secretions; animal mucus may differ from human samples; and spontaneously expectorated sputum may not represent all airway regions and may be affected by salivary contamination (Lock et al., 2018). Solids content, pH, ionic strength, rheology, collection method, storage conditions, and freeze–thaw history should be reported. A robust material should be tested across mucus samples from multiple donors and, where relevant, different disease severities, because donor variability is relevant to the intended use environment and should not be treated solely as experimental noise (Chisholm et al., 2019).

### 4.2 Oxidative and reductive gradients

Redox-active and redox-responsive systems can be divided conceptually into materials that consume reactive species, materials that release cargo after oxidative activation or cleavage, and materials that exploit intracellular reducing conditions. Antioxidant carriers may incorporate catalytic enzymes, radical-scavenging polymers, polyphenols, thiol-containing components, or inorganic catalytic domains. Oxidation-sensitive motifs include thioketals, boronic esters, peroxalate esters, thioethers, selenides, and tellurides (Wilson et al., 2010). Intracellular reduction is commonly exploited through disulfide bonds that undergo thiol–disulfide exchange in glutathione-rich environments (Oh et al., 2026).

A meaningful design must specify the relevant compartment. During airway inflammation, extracellular airway secretions may exhibit increased oxidative burden, but their redox state is dynamically buffered and varies across disease states and airway regions. The endosomal and cytosolic environments differ substantially in redox potential and glutathione availability. A carrier intended to cross mucus and enter a macrophage might use an oxidation-resistant exterior and a glutathione-responsive internal linker. Conversely, if extracellular release in inflamed mucus is intended, the activation threshold should be matched to oxidant concentrations and kinetics measured in disease-relevant airway secretions, and activation during manufacturing or storage must be avoided. Sequential logic may improve specificity but should be justified by measurements of trigger concentration and kinetics in human-relevant samples.

Response curves should be reported rather than single “with stimulus/without stimulus” comparisons. Important parameters include baseline leakage, where applicable, half-maximal activation concentration, time to release, maximum release, and, where relevant, reversibility, trigger consumption, and the fate of reaction products. These measurements should be repeated after aerosolization. An antioxidant material should also be evaluated for its biological consequences and compartmental selectivity: broad oxidant removal may protect tissue, but excessive suppression of physiological redox signaling or oxidant-dependent antimicrobial systems could impair host defense.

### 4.3 Protease-, pH-, and enzyme-responsive materials

The activities of neutrophil elastase, matrix metalloproteinases, cathepsins, selected phospholipase A_2_ isoforms, and other enzymes can be elevated in specific airway inflammatory states (Pniewska and Pawliczak, 2013; McKelvey et al., 2021). Peptide linkers or enzyme-degradable coatings can be designed as substrates for these enzymes to expose a ligand, release a drug, alter charge, or disassemble a carrier. Protease-responsive systems are attractive because protease activity is mechanistically linked to tissue injury and mucus pathology, but local enzyme activity varies substantially among patients and across disease states. Endogenous inhibitors present in airway lining fluid can also alter net enzymatic activity. Therefore, responsiveness should be calibrated using activity assays rather than total protein concentration alone.

Airway-surface-liquid pH is affected by epithelial ion transport, infection, inflammation, and local carbon dioxide, although reported disease-associated shifts are often smaller and more variable than those used in proof-of-concept release experiments. Materials that require strongly acidic conditions may respond mainly within acidic endosomal or lysosomal compartments rather than in airway lining fluid. This is not a flaw if intracellular release is the intended mechanism, but the responsive compartment should be stated. Similar care is needed for bacterial enzymes: they may enable infection-responsive release, yet enzyme expression depends on microbial species, growth state, and biofilm localization, and bacterial infection or biofilm is not present in every patient or disease state.

Dual- or multi-input logic may reduce nonspecific activation, for example, by requiring both a protease and an intracellular reducing environment. However, spatial or temporal heterogeneity of the triggers may prevent all required conditions from being met and thereby reduce effective cargo release, while complex architectures complicate characterization, manufacturing, scale-up, and quality control (López Ruiz et al., 2022). A staged development strategy is therefore generally more defensible: demonstrate that a single trigger addresses a major bottleneck, define its quantitative limitations or failure mode, and add a second trigger only if it addresses that failure.

### 4.4 Epithelial barrier dysfunction and cellular heterogeneity

The airway epithelium contains basal, ciliated, secretory cells, including goblet and club cells, tuft, neuroendocrine, ionocyte, and other specialized populations whose abundance and distribution vary along the respiratory tract (Davis and Wypych, 2021). Inflammation, smoking, infection, and remodeling can alter epithelial cell abundance, differentiation, junctional integrity, transporter expression, and innate immune signaling (Hewitt and Lloyd, 2021; Chan et al., 2022). A carrier evaluated in a single immortalized epithelial cell line therefore captures only a limited subset of relevant cell-specific uptake, barrier, and toxicity pathways.

Barrier disruption can increase apparent permeability but should not be interpreted as improved delivery. A cationic carrier may show efficient cellular uptake or transepithelial transport partly because it perturbs cell membranes or tight junctions (Mura et al., 2011). Productive uptake should be distinguished from injury using transepithelial electrical resistance (TEER), paracellular permeability tracers, junctional proteins, ciliary function where relevant, cell death, cytokine release, and recovery after exposure. Because chronic therapy may involve frequent dosing, an apparently transient change at 24 h may not predict barrier recovery or cumulative effects after repeated exposure.

Cell-selective targeting can use ligands directed to epithelial receptors or integrins and can exploit macrophage scavenger pathways or inflammation-associated markers. A central challenge is maintaining specificity after a biomolecular corona forms in airway lining fluid and mucus. Targeting ligands may be shielded, adsorbed biomolecules may alter the carrier’s biological identity, and receptor expression may vary by endotype (Mirshafiee et al., 2013). Ligand density, orientation, and accessibility should therefore be optimized rather than maximized. Active targeting can enhance cellular interaction only after a particle reaches the vicinity of the target; it cannot compensate for poor aerosol deposition or mucus trapping.

### 4.5 Extracellular matrix, mechanics, and remodeling

Chronic airway diseases modify extracellular-matrix composition and tissue mechanics. Fibronectin, collagen, proteoglycans, elastin and elastin-derived fragments, and matricellular proteins influence epithelial repair, fibroblast activation, airway smooth-muscle behavior, and immune signaling (Reeves et al., 2014). Increased matrix stiffness can reinforce profibrotic or hypercontractile phenotypes and may alter particle–cell interactions. These features are often absent from conventional submerged cultures but can be incorporated into organ-chip membranes, hydrogels, or decellularized matrices.

Mechanically responsive carriers are less established in airway delivery than redox- or enzyme-responsive systems, but the concept is relevant. Shear during nebulization may alter carrier integrity, whereas cyclic strain during breathing, mucus deformation, and local tissue stiffness may influence carrier transport or tissue responses. Irrespective of whether the carrier is mechanically responsive, mechanical context should be treated as a validation variable. A formulation that suppresses inflammatory signaling in a static monolayer may behave differently when epithelial cells experience cyclic stretch and controlled airflow. Lung-on-chip systems are particularly useful for evaluating these interactions (Liu et al., 2025). Table 1 links the major airway barriers discussed above with corresponding material strategies, intelligence-enabled tasks, human-relevant validation approaches, and the design decisions they are intended to support.

**TABLE 1:** Engineering matrix linking airway barriers to smart-material functions, computational tasks, and validation requirements.

Airway barrier or development need	Material or formulation strategy	Intelligence-enabled task	Human-relevant validation	Decision supported
Viscous mucus, plugs, and impaired clearance	Dense hydrophilic or zwitterionic shielding; controlled mucoadhesion; detachable coatings; nano-in-micro disassembly	Predict diffusion and retention from size, surface chemistry, mucus descriptors, and donor variability	Disease-derived mucus; particle tracking; mucociliary airway chip with quantified deposited dose	Penetrating versus luminal-retention design; coating density; particle size
Extracellular oxidative stress and intracellular reducing environment	Antioxidant carriers; oxidation-labile linkers; disulfide-mediated intracellular release; sequential redox logic	Estimate activation thresholds, leakage, release kinetics, and benefit-risk across redox ranges	Airway chip with extracellular and intracellular redox measurements; repeated exposure	Trigger chemistry, release compartment, dose, and safety window
Protease-rich inflammation	Elastase- or MMP-cleavable shells, linkers, or masking groups	Relate enzyme activity to activation probability and patient subgroup	Human sputum/airway fluid activity assays; inflammatory airway chip	Whether enzyme responsiveness adds value over sustained release
Epithelial barrier injury	Non-disruptive uptake; receptor targeting; endosomal escape; barrier-protective cargo	Optimize uptake while constraining permeability and toxicity	Differentiated primary epithelium; TEER/permeability; junctions; ciliary recovery	Surface charge, ligand density, and repeat-dose limits
Macrophage dysfunction or excessive particle clearance	Phagocyte-targeted carriers; biodegradable particles; phenotype-modulating cargo	Predict cell-selective uptake, intracellular release, and accumulation	Macrophage-epithelium chip; efferocytosis, phagocytosis, metabolism, cytokines	When macrophage uptake is therapeutic versus a clearance liability
Small-airway underdelivery and heterogeneous obstruction	Respirable porous particles; device-flow optimization; hygroscopic or transformable systems	CFD, reduced-order deposition, and multi-objective formulation-device optimization	Cascade impaction; realistic airway replicas; imaging-validated deposition models	Particle distribution, device, inhalation maneuver, and target region
Labile biologic or nucleic-acid cargo	Lipid/polymer protection; dry-powder stabilization; endosomal escape; intracellular release	Predict stability, aerosol stress, transfection, potency, and innate immune risk	Post-aerosol integrity; ALI/chip target engagement; repeated-dose inflammation	Carrier class, protectant, process, and clinically plausible dose
Complexity, scale-up, and batch variability	Simplified architecture; scalable mixing/drying; potency-linked quality controls	Process-property models, design space, anomaly detection, and active learning	Pilot-scale batches; device compatibility; stability; interlaboratory validation	Critical quality attributes and manufacturable operating range
Patient heterogeneity and changing disease state	Robust or adaptive formulation-device strategies	Patient-specific deposition, uncertainty propagation, longitudinal model updating	Multiple-donor chips; imaging/functional validation; prospective comparison	Whether personalization changes device, dose, or formulation choice

## 5 Material platforms for responsive inhalation

Figure 2 provides an overview of the four material families considered in this section: polymeric nanoparticles and nanocapsules, liposomes and lipid nanoparticles, inorganic/hybrid/catalytic nanomaterials, and biomimetic carriers and extracellular vesicles. The boundaries between these groups are not absolute, because hybrid and transformable systems may incorporate features of more than one platform.

**FIGURE 2:**
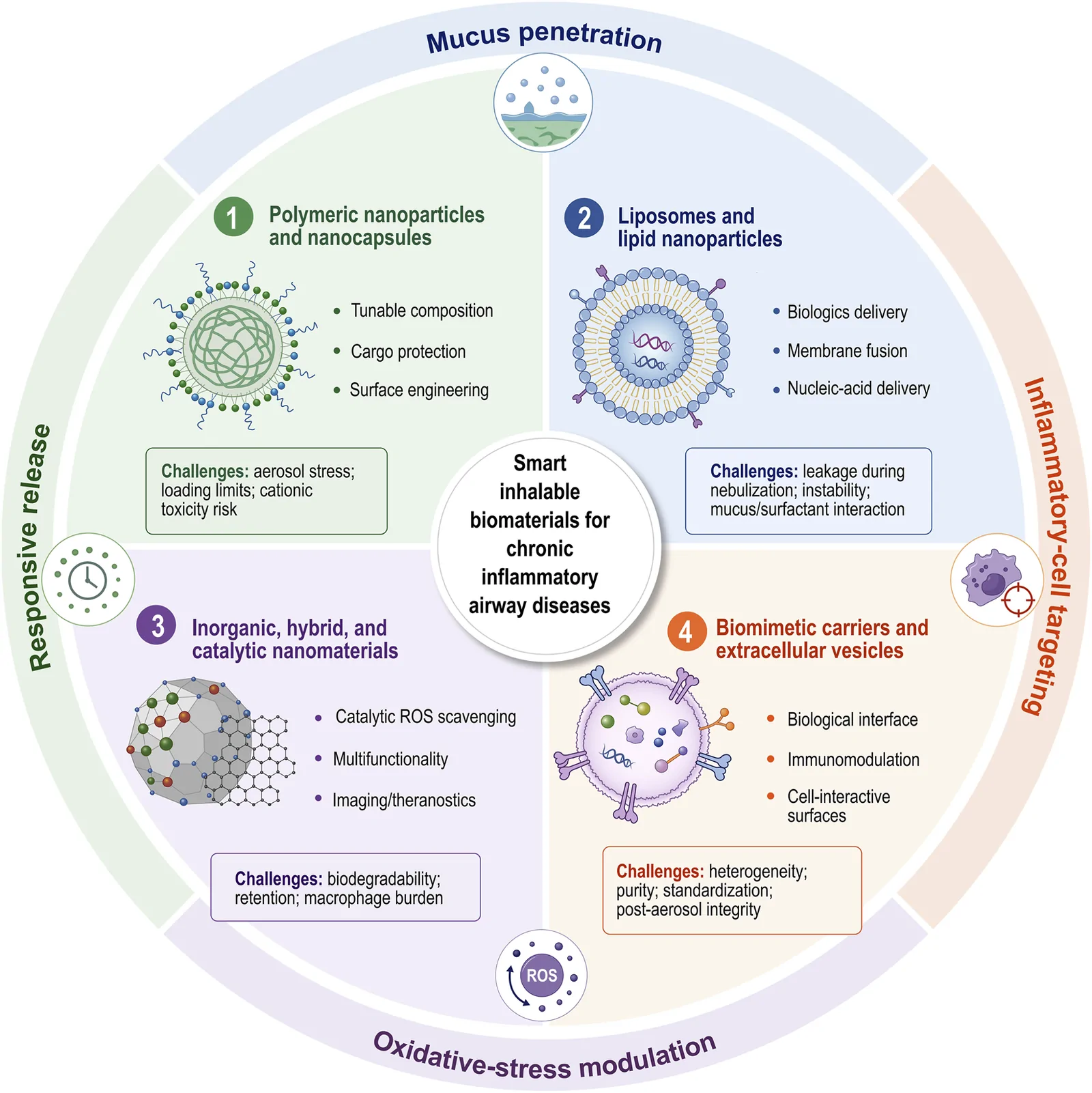
Major smart inhalable biomaterial platforms for chronic inflammatory airway diseases. Four broad platform families are illustrated: polymeric nanoparticles and nanocapsules; liposomes and lipid nanoparticles; inorganic, hybrid, and catalytic nanomaterials; and biomimetic carriers and extracellular vesicles. Each family is summarized according to representative functional advantages and major translational constraints relevant to pulmonary delivery. The outer ring highlights cross-cutting design objectives, including mucus penetration, responsive release, oxidative-stress modulation, and inflammatory-cell targeting. These functions are not exclusive to individual material classes, and a given platform may support more than one design objective depending on its composition, surface engineering, cargo, and post-deposition behavior.

### 5.1 Polymeric nanoparticles and nanocapsules

Polymeric carriers offer broad control over composition, degradation, surface chemistry, and release. Poly (lactic-co-glycolic acid) (PLGA), polycaprolactone, other polyesters, polyanhydrides, poly (β-amino ester) s, chitosan derivatives, alginate, hyaluronic acid, and synthetic responsive polymers have all been considered for pulmonary delivery (Mansour et al., 2024). Their advantages include protection of labile cargo, tunable degradation, and compatibility with surface modification. Potential limitations include residual solvents, molecular-weight dispersity and batch variability, acidic degradation products from some polyesters, limited drug loading for certain cargos or formulations, and the possibility that highly cationic polymers disrupt the airway barrier, depending on surface charge density and dose.

Nanocapsules can spatially separate an active core from a functional shell. A recent inhalable, transformable nanocapsule combined mucus penetration with catalase-mediated reactive oxygen species scavenging, illustrating how a carrier can integrate transport and antioxidant function (Zhang et al., 2025). The translational question is whether mucus penetration and reactive oxygen species-scavenging activity are preserved after aerosolization and whether catalase retains structural integrity and enzymatic activity during storage and device passage. Enzyme loading also complicates potency assays because catalytic activity, not only protein content, must be measured.

For nucleic acids, polymeric mucus-penetrating particles can protect plasmid DNA and improve gene delivery to the airway epithelium in preclinical muco-obstructive lung disease models (Kim et al., 2022). The same design principles may be adaptable to siRNA, mRNA, or genome-editing payloads, but endosomal escape, innate immune activation, and intracellular trafficking remain important, cargo-dependent constraints. A surface that is inert enough to cross mucus may be inefficient for cell entry, which may motivate detachable shielding or pH-responsive internal components that become active after cellular uptake.

### 5.2 Liposomes and lipid nanoparticles

Liposomes and lipid nanoparticles have established advantages for encapsulating hydrophobic and amphiphilic drugs, proteins, and nucleic acids. Their lipid components can be selected to promote membrane fusion, endosomal escape, mucus penetration, or tune biodegradability. Pulmonary delivery, however, introduces challenges distinct from intravenous administration. Serum stability is less immediately dominant before systemic absorption, whereas interactions with mucus, pulmonary surfactant, airway lining fluid, and aerosol interfaces become central. Ionizable lipids optimized for systemic mRNA delivery may not provide optimal pulmonary tolerability or compatibility with mucus and surfactant and may require route-specific reformulation (Lokugamage et al., 2021).

Mucus-penetrating lipid nanoparticles have enabled inhaled delivery of mRNA payloads to alveolar epithelial cells in a preclinical pulmonary fibrosis model (Wang et al., 2024). Although pulmonary fibrosis differs from asthma and COPD, the study demonstrates that route-specific lipid and surface engineering can support mucus penetration and epithelial mRNA expression after inhalation. Translation to chronic inflammatory airway diseases would require matching the target cell, characterizing and minimizing unintended innate immune activation, and evaluating repeated administration. For type 2 inflammation, possible nucleic-acid cargos could modulate epithelial alarmins, mucin production, or cytokine signaling; for COPD, potential targets may include oxidative stress, senescence, macrophage function, or protease balance. These possibilities remain preclinical and should not be presented as established therapies.

Lipid composition also affects aerosol stability. Nebulization can cause cargo leakage, aggregation or fusion, lipid oxidation, or changes in particle size, depending on the formulation and device. Dry-powder conversion may improve storage stability but generally requires stabilizing excipients or matrix formers and verification of nanoparticle recovery after redispersion. The formulation should be characterized before and after device passage, including lipid integrity, cargo encapsulation and integrity, biological potency, particle-size distribution, and aerosol performance.

### 5.3 Inorganic, hybrid, and catalytic nanomaterials

Inorganic nanomaterials, depending on their composition and structure, can provide catalytic antioxidant activity, imaging contrast, magnetic responsiveness, or photothermal function. Cerium oxide, manganese dioxide, iron oxide, silica, gold, and other materials have been explored in pulmonary contexts. Their low solubility or slow degradation may be advantageous for some functions but raises concerns about long-term retention and prolonged macrophage sequestration or overload (Kreyling et al., 2018). For chronic airway diseases, dissolution, degradability where applicable, and pulmonary clearance are particularly important because repeated administration may be required.

Hybrid nanomaterials combine polymers, lipids, proteins, cell membranes, or inorganic domains to integrate complementary functions. An engineered hybrid mucus-penetrating nanomedicine has shown enhanced lesion accumulation and therapeutic delivery in a preclinical model of idiopathic pulmonary fibrosis (Zou et al., 2025). Hybridization may improve cargo loading, transport, or biological functionality, but it also increases the number of critical quality attributes. Material interfaces may be altered during manufacturing, aerosolization, storage, or interaction with airway fluids. A hybrid system should therefore be justified by a measurable performance gain that is not readily achieved with a simpler platform.

Catalytic nanomaterials should be evaluated under physiologically relevant substrate concentrations and in the presence of airway proteins and antioxidants. Catalytic activity measured in a simple buffer may be reduced by biomolecular adsorption, restricted substrate access, or local pH shifts. Potential interference with oxidant-dependent host-defense chemistry should also be assessed. Long-term biodistribution and clearance studies are particularly important for poorly degradable catalytic particles.

### 5.4 Biomimetic carriers and extracellular vesicles

Cell-membrane coating and extracellular-vesicle-based delivery seek to leverage natural biological surface features, intercellular communication mechanisms, reduced immune recognition, or cell-specific interactions. Membranes derived from macrophages, neutrophils, platelets, epithelial cells, or engineered source cells can present complex combinations of surface proteins that are not readily reproduced by a single synthetic ligand. Extracellular vesicles can carry proteins, lipids, and nucleic acids and may have source- and state-dependent immunomodulatory activity (Welsh et al., 2024).

The complexity of these systems is both their advantage and a major translational obstacle. Membrane composition varies with cell source, activation state, culture conditions, passage where applicable, and membrane-isolation or EV-separation method. Extracellular-vesicle preparations can contain non-vesicular proteins, lipoproteins, unencapsulated or co-isolated nucleic acids, and media-derived contaminants. Potency may depend on multiple incompletely defined components. For inhalation, aerosol shear and air–liquid interfaces may alter vesicle integrity, cargo retention, or membrane-associated proteins in a device- and formulation-dependent manner (Han et al., 2022). Standardization therefore requires assessment of source identity, membrane or vesicle integrity, EV-associated and non-EV markers, purity, particle concentration and protein content reported separately, functional potency assays, and post-aerosol characterization.

Biomimetic systems may be attractive for inflammatory-cell targeting, but apparent homing should be distinguished from local phagocytic uptake and, where systemic exposure occurs, capture by the mononuclear phagocyte system. In airway disease, mucus components and pulmonary surfactant can interact with the carrier surface and may alter its biological identity or reduce the accessibility of biologically derived ligands. Patient-derived mucus and organ-chip models can help determine whether the expected surface accessibility and intended cell interactions are preserved in the actual delivery environment.

### 5.5 Protein, peptide, antibody, and nucleic-acid cargos

Smart materials expand inhalation beyond conventional small molecules. Proteins, antibodies, peptides, siRNA, antisense oligonucleotides, mRNA, plasmid DNA, and gene-editing payloads could act on targets that are difficult to modulate with conventional inhaled small-molecule drugs. Local delivery may reduce systemic exposure and, for locally acting agents, lower total dose requirements, but these cargos may be sensitive to different formulation and aerosolization stresses, including shear, interfacial stress, oxidation, proteolysis, or nuclease degradation, depending on the cargo and formulation. Many also have limited epithelial permeability when intracellular or transepithelial delivery is required, and some may activate innate immune pathways.

For proteins and antibodies, formulation must preserve higher-order structure and binding activity. For nucleic acids, protection from extracellular nucleases, mucus penetration, cellular uptake, endosomal escape, and cytosolic or nuclear delivery, depending on the cargo and its site of action, are required. Each added barrier can reduce overall efficiency, making dose accounting essential. A high transfection percentage in cells exposed directly to a nanoparticle suspension does not necessarily predict airway gene transfer after aerosolization. More informative experiments directly expose differentiated airway tissue to the emitted aerosol and measure spatial transgene expression, inflammatory responses, and tissue recovery during and after repeated exposure (Hennig et al., 2025).

### 5.6 Nano-in-micro and transformable systems

Nano-in-micro systems couple nanoparticle-level biological function with micron-scale aerosol engineering. Nanoparticles are embedded in or associated with respirable microparticle matrices produced by spray drying, spray-freeze drying, thin-film freezing, or related particle-engineering processes (Jiang et al., 2022). Excipients such as leucine, mannitol, trehalose, and lactose can improve powder dispersibility, stabilize nanocarriers, or protect cargo, depending on their physicochemical properties, while polymeric matrix formers such as alginate can additionally provide structural or release-controlling functions. After deposition, the microparticle matrix is intended to dissolve or disintegrate, releasing the nanocarrier.

These excipients play distinct and non-interchangeable roles in nano-in-micro formulations. Leucine functions primarily as a surface-active dispersibility enhancer: during spray drying, it can preferentially enrich and crystallize at the particle surface, reducing interparticle cohesion and moisture sensitivity while promoting surface corrugation and favorable aerosolization. Mannitol acts mainly as a water-soluble crystalline bulking and matrix-forming excipient that provides structural support to the dried microparticle; however, its crystallization and polymorphic state can interact with leucine surface enrichment and thereby influence particle morphology, density, and aerodynamic performance (Schweizer et al., 2024). Alginate serves a different function as a hydrophilic polymeric matrix former. Its gel-forming and mucoadhesive properties can improve payload entrapment and support sustained release, but increasing polymer content may also modify particle morphology, dissolution, and aerosol performance, making its concentration and matrix architecture important formulation variables (Alizadeh et al., 2024). Excipient identity, ratio, and solid-state behavior should therefore be treated as design variables rather than as interchangeable stabilizers.

This architecture creates at least three interdependent optimization levels: dry-powder particle properties, aerosol behavior, and post-deposition nanoparticle function. The formulation should minimize irreversible aggregation, premature cargo leakage, or surface-property changes during drying. Residual moisture and solid-state transitions can influence storage stability (Keil et al., 2021). A powder may show excellent aerosol performance but release aggregated nanoparticles with poor mucus transport. Conversely, a nanoparticle with favorable biological properties may be difficult to formulate at a therapeutically useful payload while retaining acceptable aerosol performance. Intelligence-enabled multi-objective optimization is well suited to this problem because no single metric defines success.

Transformable systems extend the concept by intentionally changing size, charge, deformability, or structure after pulmonary deposition or upon entry into the target compartment. A microparticle may release nanoparticles; a mucus-inert coating may detach to expose a cell-interactive surface; or a carrier may change size after enzymatic cleavage. Transformation should be demonstrated after aerosolization in disease-relevant fluids, with kinetics matched to mucus transit, target-site exposure, cellular uptake, and clearance (Zhu et al., 2024). Transformation that occurs before the intended target region is reached may cause premature cargo release in proximal airways, whereas overly slow transformation may result in removal before activation.

### 5.7 Choosing between mucus penetration, retention, and cellular uptake

A rational platform begins with a map of the intended delivery route. For an intracellular epithelial target, the sequence may be aerosol deposition, mucus diffusion, epithelial contact, endocytosis, endosomal escape, and intracellular release. For an extracellular antiprotease, the desired route may end in the mucus layer. For macrophage reprogramming, deposition in the compartment containing the target macrophage population and phagocytic uptake may be beneficial, while epithelial uptake or transepithelial transport is less important. Material requirements should be derived from this route rather than selected from a generic list of desirable nanoparticle properties.

Trade-offs are common. Dense hydrophilic shielding can improve mucus penetration but may reduce cellular interaction. Positive surface charge can enhance cell association but may also increase mucus binding and membrane perturbation, depending on charge density and dose. Larger or denser aerosol particles may carry more payload per particle but are more susceptible to proximal impaction. Strong mucoadhesion may increase local interaction but can also promote removal with physiologically cleared mucus. Slow degradation may extend exposure but raises accumulation risk. These trade-offs support Pareto-based design rather than a search for a single globally optimal formulation. Table 2 compares these platforms in terms of cargo compatibility, major advantages and limitations, aerosol and device considerations, and the applications for which each is best suited.

**TABLE 2:** Comparison of major inhalable biomaterial platforms that can support smart functions in chronic inflammatory airway diseases.

Platform	Typical cargos	Major advantages	Main limitations	Device/aerosol considerations	Best-fit application scenario
Polymeric nanoparticles	Small molecules; proteins; nucleic acids	Tunable composition; surface engineering; cargo protection	Loading constraints; batch variability; cationic toxicity risk	Must retain size and function after nebulization or drying	Mucus penetration and intracellular epithelial delivery
Nanocapsules	Fragile enzymes; antioxidants; nucleic acids	Spatially separated core-shell function; transformable design	More complex characterization; shell instability risk	Need preserved shell integrity during aerosolization	Responsive or sequential delivery
Liposomes/lipid nanoparticles	Hydrophobic drugs; proteins; mRNA; siRNA	Biocompatibility; membrane fusion; biologics delivery	Leakage; fusion; surfactant and mucus interaction	Nebulization may alter size and encapsulation	Biologic or nucleic-acid pulmonary delivery
Inorganic/catalytic nanomaterials	Catalytic or imaging functions	ROS scavenging; imaging contrast; multifunctionality	Slow degradation; retention; macrophage burden	Repeated-dose safety and clearance are critical	Oxidative-stress modulation and theranostics
Hybrid nanomaterials	Drugs; proteins; nucleic acids; catalytic domains	Combines complementary functions	More critical quality attributes; manufacturing complexity	Interfaces may change during storage or aerosol passage	Use when a measurable gain over simpler systems is justified
Biomimetic membrane-coated carriers	Small molecules; proteins; nucleic acids	Complex biological surface; improved cell interaction	Source variability; surface masking in mucus	Post-aerosol membrane integrity must be verified	Inflammatory-cell targeting
Extracellular vesicles	Endogenous proteins; lipids; RNAs; loaded therapeutics	Natural intercellular communication; immunomodulatory potential	Purity; heterogeneity; potency standardization	Shear and air-liquid interfaces may alter vesicle integrity	Biologically active delivery and immunomodulation

Material class alone does not determine whether a carrier is considered “smart.” The platforms listed here are included because they can be engineered to support stimulus-responsive, transformable, context-selective, or other programmed functions. Conventional formulations lacking such functions are not classified as smart inhalable biomaterials under the operational definition used in this review.

## 6 Intelligence-enabled material and formulation development

### 6.1 Building structured and reusable datasets

Machine learning cannot compensate for poorly defined or poorly documented data. Inhalable-biomaterial datasets assembled across studies may combine experiments performed with different devices, impactors, flow rates, mucus models, cell systems, and analytical methods. Incomplete metadata and the omission of failed or underperforming formulations can limit comparison and reuse. Before choosing an algorithm, the field needs structured descriptors and consistent, standardized metadata.

Dataset fields can be organized into material descriptors, process descriptors, aerosol descriptors, biological-context descriptors, and patient- and geometry-specific descriptors. Material descriptors include composition, molecular weight, lipid ratios, surface functional-group density, morphology, geometric size, particle density, charge, ligand density, cargo properties, and solid-state properties. Process descriptors include mixing order, solvent, energy input, temperature, drying conditions, feed concentration, atomization conditions, and storage conditions. Aerosol descriptors include device type, device resistance, flow waveform, emitted dose, MMAD, GSD, FPF, and plume properties. Biological-context descriptors include mucus solids content, rheology, donor characteristics, cell source, differentiation state, inflammatory stimulus, mechanical strain, and exposure dose. Without these contextual variables, models risk learning laboratory- or protocol-specific signatures rather than generalizable structure–performance relationships (Jiang et al., 2022).

A minimum reporting schema could make historical data more reusable. Controlled vocabularies and data dictionaries should distinguish loaded dose from deposited dose and hydrodynamic size from aerodynamic size. Units should be standardized, and measurement methods and conditions should be recorded explicitly. Raw distributions should be retained where possible because averages can conceal distributional heterogeneity and outliers. Images, such as scanning electron micrographs, can be linked to tabular process data, while time-dependent release and barrier data should be represented as complete time series, curves, or appropriately defined curve-derived parameters rather than single endpoints.

### 6.2 Supervised learning for structure-property-performance mapping

Supervised learning can predict particle size, encapsulation efficiency, stability, aerosol performance, mucus diffusion, cell uptake, toxicity, or release kinetics from formulation and process variables. Thin-film-freezing studies have demonstrated prediction of dry-powder aerosol performance using tabular variables and image information (Jiang et al., 2022). More recent work has applied machine learning to inhalable antibiotic dispersions (Bahjat et al., 2025) and spray-dried anti-tuberculosis formulations (Hu et al., 2026). These examples show feasibility, but most datasets remain small and local.

Algorithm choice should reflect sample size and objective. Linear or regularized models can be robust and interpretable for limited data. Tree-based ensembles capture nonlinear interactions and mixed variable types. Gaussian processes provide uncertainty estimates and are useful for Bayesian optimization. Neural networks may be valuable for images or large datasets but can overfit small formulation studies. Performance should be evaluated with nested cross-validation or an independent test set, and splits should avoid leakage from replicate batches or near-identical formulations.

Feature importance must be interpreted cautiously. Correlated variables can share or distort importance, and a high predictive contribution does not establish causality. SHAP values, partial dependence, and accumulated local effects can help explore model behavior, but mechanistic experiments are needed to confirm a proposed relationship. The most useful model is one that suggests a testable formulation decision, not merely one that reports a high coefficient of determination.

### 6.3 Multi-objective optimization and pareto design

Inhaled biomaterials typically require consideration of multiple competing objectives, including drug loading, encapsulation efficiency, stability, respirable fraction, mucus penetration, retention, uptake, release, potency, safety, manufacturability, and cost. Combining these outputs into a single weighted score may obscure clinically important trade-offs. Pareto optimization instead identifies non-dominated formulations for which improvement in one objective requires a sacrifice in at least one other objective. This allows investigators to compare, for example, a high-payload formulation with moderate mucus transport against a lower-payload formulation with better distal deposition or distribution.

Hard constraints should be separated from objectives. Sterility, endotoxin limits, cytotoxicity above a predefined acceptance threshold, or failure to meet a predefined aerodynamic-performance range may define exclusion criteria rather than variables to be traded. The remaining feasible formulations can then be prioritized according to context-specific requirements. A formulation for maintenance therapy may prioritize repeated-dose safety and stability, whereas one for acute exacerbation may place greater emphasis on rapid onset and device usability in patients with reduced inspiratory capacity.

### 6.4 Active learning and Bayesian optimization

Conventional design of experiments samples a predefined space efficiently but may still require many experiments when interactions are complex. Active learning and Bayesian optimization select subsequent experiments based on the current model, with Bayesian optimization explicitly balancing exploration of uncertain regions against exploitation of promising formulations through an acquisition function. These approaches are attractive when each aerosol or organ-chip experiment is costly or time-consuming.

An effective loop begins with a diverse initial design, fits a probabilistic surrogate, selects new candidates according to an acquisition function, performs experiments, and updates the model. Batch selection can be used to match laboratory throughput. Importantly, failed or underperforming formulations should remain in the dataset because they help define feasibility and performance boundaries (Narayanan et al., 2021). Human oversight is required to prevent the algorithm from repeatedly proposing chemically infeasible or clinically irrelevant candidates. Physicochemical and translational constraints, such as charge balance, solubility, or maximum excipient exposure, can be incorporated into the design space.

### 6.5 Hybrid mechanistic-data-driven models

Purely empirical models can perform well within the domain represented by their training data but may lose accuracy when applied to a new device, material class, or patient population. Mechanistic models encode conservation laws, aerosol physics, diffusion, dissolution, reaction kinetics, or cellular transport and may support extrapolation when their assumptions, parameters, and boundary conditions remain valid. Hybrid models seek to combine these strengths. For example, a mechanistic or CFD-based deposition model can generate regional exposure data, while a learned surrogate accelerates repeated prediction. A release model can be constrained by mass balance while machine learning infers uncertain rate parameters.

Physics-informed neural networks (PINNs) are one possible implementation, but the broader principle is more important than the architecture. Known relationships need not be relearned solely from small datasets when they can be encoded explicitly. Mechanistic assumptions must still be validated; a detailed model with uncertain boundary conditions may create an appearance of false precision. Sensitivity and uncertainty analyses can identify influential parameters, assumptions, and sources of predictive uncertainty and thereby help prioritize measurements that may improve confidence (Saltelli et al., 2019).

### 6.6 Image and time-series intelligence

Material development and validation generate images of particle morphology, aerosol plumes, mucus transport, tissue architecture, and organ-chip responses. Computer vision and quantitative image analysis can quantify features that are difficult to capture manually, including particle shape, surface texture or apparent porosity, aggregation, ciliary motion, mucus velocity, barrier defects, and spatial cytokine or cell-state patterns where supported by the imaging modality (Oldenburg et al., 2012). For heterogeneous imaging inputs, multimodal fusion architectures, including cross-attention-based approaches, may further integrate complementary information across imaging sources before downstream prediction or classification (Sreekala et al., 2026). Time-series analysis can characterize release profiles, barrier recovery, breathing cycles, and longitudinal connected-inhaler signals (Tsang et al., 2023).

Image models are vulnerable to batch, site, instrument, and acquisition-protocol effects. Magnification, contrast, staining, microscope or scanner type, and preprocessing may become shortcut features rather than biologically or materially meaningful signals (Homeyer et al., 2022). External validation across laboratories, instruments, and acquisition protocols is therefore important when cross-site or cross-device generalizability is claimed. Whenever possible, interpretable morphological features should be extracted and linked to process variables alongside learned image features, rather than relying only on black-box image predictions.

### 6.7 Uncertainty, calibration, and domain of validity

A point prediction without an accompanying uncertainty estimate is difficult to use for experimental selection or patient-specific decisions. Uncertainty can arise from measurement error, donor variation, batch variation, incomplete descriptors, model structure, and distribution shift caused by out-of-domain inputs. Ensembles, Gaussian processes, Bayesian models, conformal prediction, and bootstrapping can quantify different aspects of predictive uncertainty under different assumptions. Calibration should be assessed so that predicted probabilities correspond to observed frequencies and the empirical coverage of prediction intervals approaches their nominal coverage.

Models should also assess whether a candidate lies outside the training domain. A model trained only on polymeric nanoparticles should not return an unqualified prediction for a lipid nanoparticle or extracellular vesicle. Similarity metrics, applicability-domain rules, and explicit abstention or “do not predict” outputs are preferable to unsupported extrapolation with high apparent confidence. For patient-specific deposition, uncertainty in image segmentation, airway truncation, boundary conditions, and inhalation waveform should, where feasible, be propagated into regional dose estimates (Sadafi et al., 2024a).

### 6.8 Explainability, reproducibility, and data governance

Explainability serves at least two distinct purposes. Scientific explainability asks whether the model suggests plausible relationships that can be tested experimentally. Operational explainability asks whether a developer or clinician can understand why a formulation, device, or dose was recommended. These needs may be addressed with simpler surrogate models, sensitivity analysis, counterfactual examples, or transparent rule-based constraints, selected according to the user and decision context, rather than a single global explanation method (Antoniadi et al., 2021).

Reproducibility requires versioned data, code, preprocessing, model parameters, random seeds, and prospective testing. Proprietary formulation data create additional barriers, but federated or privacy-preserving learning may allow multi-institutional models without centralizing sensitive data. Data governance should define ownership, access, quality control, and responsibility for model updates (Bhasha and Panda, 2024). In a regulated development context, changes to an algorithm or dataset can change the product-development decision and therefore require documentation.

## 7 Lung-on-chip validation of inhaled biomaterials

### 7.1 Why static models are insufficient

Submerged epithelial monocultures are useful for screening but do not reproduce, in an integrated manner, the air-exposed epithelial surface, mucus, mucociliary differentiation, ciliary activity, vascular interface, immune-cell trafficking, and mechanical strain present in the human lung. Directly adding a nanoparticle suspension to culture medium bypasses aerosol generation and alters the biomolecular corona, delivered concentration, sedimentation, and cellular uptake pathway relative to aerosol exposure (Bessa et al., 2021). It can therefore misrepresent effective exposure and obscure device- and aerosolization-related failure.

Air–liquid interface cultures can improve epithelial differentiation and allow apical aerosol exposure, but conventional inserts remain largely static and provide limited control over continuous perfusion and mechanical strain. Lung-on-chip systems can incorporate microfluidic flow, tissue–tissue interfaces, cyclic stretch, defined extracellular-matrix environments, integrated sensing, and real-time imaging. The foundational lung-on-chip reproduced key features of the alveolar–capillary interface and breathing-like cyclic mechanical strain (Huh et al., 2010), and a small-airway chip subsequently modeled inflammation and drug responses using differentiated human small-airway epithelium and microvascular endothelium (Benam et al., 2016). These platforms do not replace all animal or clinical studies, but they can help address critical gaps between material characterization and *in vivo* translation.

### 7.2 Essential components of an airway-relevant chip

A chip for asthma or COPD should be selected according to the biological question and target compartment. A conducting-airway model may include differentiated primary bronchial epithelial cells forming a mucociliary epithelium at an air–liquid interface, an endothelial compartment, extracellular matrix, and controlled airflow where relevant (Benam et al., 2016). An alveolar model may include alveolar epithelial cells, microvascular endothelium, surfactant, and breathing-like cyclic strain. A small-airway model may incorporate small-airway-scale geometry, distal epithelial phenotypes, and disease-specific cells.

Cell source strongly influences model relevance. Immortalized lines can improve reproducibility and throughput but may have limited differentiation. Primary cells can retain donor- and disease-associated phenotypes but vary between donors and may lose characteristics during expansion. Induced pluripotent stem cell-derived cells offer scalability and genetic tractability but require rigorous maturity assessment. Organoid-derived epithelia and COPD organoids provide additional patient-relevant options (Seo et al., 2022). A tiered strategy can use standardized lines for early screening and primary or patient-derived cells for confirmation.

The extracellular matrix and membrane also matter. Polydimethylsiloxane (PDMS) is common but can absorb or sequester some hydrophobic small molecules and thereby alter their free concentration. Alternative low-sorption materials can mitigate this limitation. Selected thermoplastic elastomers, including styrenic block-copolymer-based systems, can retain mechanical flexibility while exhibiting lower small-molecule absorption than PDMS, whereas cyclic olefin copolymers/polymers (COC/COP) provide optically transparent, chemically stable thermoplastic platforms with substantially reduced sorption of many lipophilic compounds (Schneider et al., 2021; Grindulis et al., 2025). Material selection nevertheless involves trade-offs in flexibility, gas permeability, bonding, manufacturability, and surface properties, and should therefore be matched to the biological and pharmacological requirements of the chip. Synthetic porous membranes may be thicker or stiffer than native basement membranes. Hydrogels can improve extracellular-matrix mimicry but may complicate fabrication, imaging, and standardization (Corral-Nájera et al., 2023).

### 7.3 Coupling aerosol exposure to the chip

A major advance in inhaled-material evaluation is direct exposure of the tissue to a device-generated aerosol. A multiplex platform has coupled aerosol generation to a breathing lung-on-chip, enabling in situ-like aerosol exposure (Sengupta et al., 2023). Dynamic breathing chips have shown that mechanical motion can influence inhaled-drug response and cellular uptake (Liu et al., 2025). A breathing mucociliary-on-a-chip system coupled to inhalation exposure has further enabled visualization and quantification of aerosol deposition, nanodrug penetration, and mucociliary clearance. These studies support a shift from nominal suspension dose toward device-generated and tissue-deposited exposure.

Aerosol–chip coupling requires careful dosimetry. The nominal amount loaded in a nebulizer, the airborne aerosol concentration in an exposure chamber, and the mass deposited on the tissue are different. Depending on the aerosol and analyte, quartz crystal microbalances, fluorescence, chemical assays, radiolabeling, or gravimetric or collected-mass measurements can be used to quantify deposition (Ding et al., 2020). The spatial uniformity of deposition across the chip should be assessed because a uniform chamber concentration does not guarantee a uniform tissue dose. Evaporation and changes in droplet composition before deposition can alter droplet size and the solute concentration delivered to the tissue.

The exposure system itself can introduce size-dependent transmission losses or alter formulation integrity. Tubing length and bends, electrostatic deposition, condensation, and exposure-chamber geometry should be characterized where present. For dry powders, reproducible dispersion and delivery to the chip remain technically challenging but important. A defensible study reports device settings, airflow conditions, aerosol size distribution at the chip inlet, deposited mass, and, where applicable, carrier integrity after aerosol generation and deposition.

### 7.4 Modeling mucus and mucociliary clearance

A mucus-relevant chip should aim to reproduce mucus secretion, hydration, ciliary beating, and directional transport rather than relying solely on an externally added static mucin layer. Depending on the disease question, modeling can use IL-13 to induce goblet-cell metaplasia and mucus production, cigarette smoke to impair epithelial function, inflammatory cytokines, microbial products, altered hydration, or patient-derived cells. A high-throughput bronchus-on-chip system has demonstrated the feasibility of modeling inflammatory epithelial changes in a plate-based format (Mori et al., 2024).

Relevant readouts may include mucus thickness, mucin composition, mucus solids concentration, rheology, ciliary beat frequency, ciliary beat coordination, transport velocity, plug formation, particle diffusion, and mucociliary clearance. Combining live particle tracking with epithelial and inflammatory readouts can help determine whether improved penetration translates into greater tissue exposure or accelerated mucociliary removal without increased tissue exposure. Repeated-exposure studies are important for formulations intended for chronic or repeated administration because some materials may alter mucus production or ciliary function over time.

### 7.5 Barrier transport, immune recruitment, and target engagement

Barrier function can be measured through transepithelial electrical resistance where technically applicable, permeability tracers, junctional proteins, epithelial injury markers, and recovery kinetics. A vascular channel enables assessment of translocation into the vascular compartment as a surrogate for potential systemic exposure. Immune cells can be introduced or perfused to study recruitment, transmigration, phagocytosis, cytokine networks, and, when apoptotic target cells are included, efferocytosis. For asthma, eosinophils, mast cells, dendritic cells, or type 2 cytokine conditions may be selected according to the biological question; for COPD, macrophages, neutrophils, cigarette-smoke exposure, bacterial products, or senescence-associated states may be prioritized (Nawroth et al., 2020).

Target engagement should be distinguished from a nonspecific reduction in inflammatory readouts. If a material is designed to silence a gene, the chip should measure knockdown in the intended cell population and the relevant downstream functional consequence. If it is intended to scavenge extracellular ROS, extracellular and intracellular redox compartments should be measured separately using compartment-appropriate assays. If it reprograms macrophages, surface markers alone are insufficient; functional readouts such as phagocytosis, cytokine responses, efferocytosis, metabolism, and interactions with the epithelium should be selected according to the intended mechanism.

### 7.6 Repeated-dose safety and recovery

Products intended for chronic inhalation require repeated-dose evaluation. Chips can expose tissue repeatedly while monitoring barrier function, ciliary behavior, inflammatory adaptation, material accumulation, and recovery (Heringa et al., 2020). When non-destructive or minimally invasive readouts are available, this provides an advantage over exclusively destructive endpoint assays. The dosing schedule should reflect expected clinical use and include washout periods where clinically relevant. Material accumulation in cells and extracellular matrix, as well as retention in device components, should be quantified and incorporated into mass-balance analysis.

Safety assessment should be selected according to the material class, intended mechanism, and exposure scenario and may include cytotoxicity, oxidative stress, DNA damage, inflammasome activation, complement activation where relevant, cytokine release, excessive macrophage uptake or functional impairment, surfactant interactions, fibrosis-related signaling, and impaired host defense. Sterility and endotoxin control are essential because low-level contamination can be mistaken for material-induced inflammation (Li et al., 2017). For biologically derived carriers, unintended residual cellular components, free or co-isolated nucleic acids and proteins, and media-derived contaminants require additional characterization and control.

### 7.7 Patient-specific chips and response heterogeneity

Primary cells from multiple donors allow response heterogeneity to be quantified rather than obscured by population averaging. Donors can be stratified by asthma endotype, COPD phenotype, smoking history, exacerbation frequency, age, or treatment response, provided that these variables are defined prospectively and adequately represented. An apparent mean effect may be driven by a subset of donors, which could motivate biomarker-guided development but would require independent confirmation. However, donor numbers in organ-chip studies may be limited; statistical designs should treat the donor as the independent biological unit and technical chips from the same donor as nested or repeated observations rather than independent donor-level replicates.

Patient-specific chips are sometimes described as biological twins, but the term should be used carefully. Cells sampled from a patient capture selected biological properties at a particular time point; they do not reproduce the entire lung or its longitudinal state. Their current strength is to provide individualized *in vitro* biological-response data that can complement patient-specific physical deposition models.

### 7.8 Standardization and limitations

Organ-chip platforms vary in geometry, materials, cell source, applied strain, flow conditions, and readouts. This flexibility is useful for discovery but complicates cross-study comparison. Minimum reporting should include, where applicable, device dimensions, membrane or matrix composition, cell source and donor information, differentiation markers, flow conditions, strain amplitude and frequency, air–liquid-interface duration, mucus properties, exposure system, deposited dose and dosimetry method, and assay timing (Piergiovanni et al., 2021). Reference drugs and benchmark materials can support interlaboratory comparison.

Current limitations include limited throughput in many platforms, cost, bubble formation, variable cell differentiation, incomplete immune complexity, absorption or adsorption of compounds by device materials, and incomplete representation of neural, lymphatic, and systemic interactions (Low et al., 2021). Chips should therefore answer defined questions rather than be claimed to reproduce the entire disease. Their translational value is strengthened when they test a bottleneck that conventional systems cannot address, such as aerosol deposition under breathing, mucus clearance, or tissue response to a quantified regional dose.

## 8 Patient-specific pulmonary modeling and the path toward digital twins

### 8.1 Image-based airway reconstruction

Computed tomography can be segmented to generate patient-specific airway geometries, typically resolving the central and a portion of the conducting airway tree (Bordas et al., 2015). If distal-airway representation is required, branches below imaging resolution must be represented through algorithmic extension, reduced-order models, or compartmental assumptions. Segmentation errors, airway truncation, respiratory phase, reconstruction kernel, and intraluminal mucus can alter the reconstructed geometry. Quality control is therefore essential before simulation.

Computed tomography and quantitative image analysis can also provide lobar volumes, airway wall thickness, emphysema distribution, mucus plugs, and ventilation surrogates. These features can inform boundary conditions and identify target regions. Longitudinal CT may show changes in airway caliber or mucus burden, but repeated computed tomography raises radiation and cost considerations. Magnetic resonance imaging, spirometry, and oscillometry may provide complementary physiological information, while connected-inhaler data may provide longitudinal information on treatment use and inhaler behavior (Young et al., 2018).

### 8.2 Computational fluid dynamics and particle transport

Computational fluid dynamics solves airflow within reconstructed geometry and tracks particles by accounting for inertial impaction, aerodynamic drag, gravitational settling, Brownian diffusion, and turbulent dispersion and, where relevant, hygroscopic growth or electrostatic forces, depending on particle size and model scope. High-fidelity models can reveal regional deposition and local hotspots, but they are computationally expensive and depend on geometry, boundary conditions, and model assumptions. Recent studies have validated CFD deposition estimates against SPECT in the same population (Sadafi et al., 2024b) and developed faster approaches for total and lobar deposition (Sadafi et al., 2024a). A recent multiphysics model further demonstrated the influence of upper-airway geometry, soft-palate mechanics, thermal conditions, breathing regime, and particle properties on aerosol deposition in a realistic human upper-airway model (Issakhov et al., 2026).

For formulation development, model outputs should be connected to actionable formulation or device decisions. Useful comparisons include device plume characteristics, particle-size distribution, inhalation flow, breath hold, hygroscopic growth, or changes in airway obstruction. When input variability and parameter uncertainty are explicitly represented, the output may be a probability distribution of dose to a target lobe, airway region, or generation rather than a single deterministic map. Validation can use *in vivo* deposition imaging or *in vitro* airway replicas, whereas cascade-impactor data are better used to characterize the inlet aerosol and pharmacokinetic measurements can evaluate downstream exposure predictions when deposition is coupled to absorption and disposition models.

### 8.3 Reduced-order and surrogate models

Full CFD is often impractical for optimizing thousands of formulations or providing real-time or near-real-time clinical feedback. High-fidelity patient-specific simulations may require substantial preprocessing for image segmentation and airway reconstruction, mesh generation, specification of physiologically appropriate boundary conditions, and repeated numerical simulations of airflow and particle transport across alternative devices, particle-size distributions, or inhalation maneuvers. Depending on model resolution, geometric complexity, and available computing resources, these steps may require substantially longer than the time available for point-of-care decision-making. Computational cost is therefore not merely a technical limitation but an important determinant of whether patient-specific deposition modeling can be integrated into clinical workflows. Reduced-order models, response surfaces, one-dimensional or graph-based airway models, and machine-learning surrogates can approximate deposition more rapidly (Kuprat et al., 2025). Data-driven surrogates can be trained on high-fidelity simulation and then evaluated against independent geometries. Computational speed should not come at the expense of uncertainty reporting; reduced-order and surrogate models require a defined applicability domain and quantified prediction error.

A hierarchical-fidelity strategy can be useful. Early screening may use population-average or idealized models, intermediate development may use a set of representative disease geometries, and late-stage evaluation may use patient-specific models. High-fidelity CFD can generate training data for a fast surrogate that is embedded in a multi-objective formulation search. Prioritized candidate formulations selected by the surrogate can then be re-evaluated with high-fidelity simulation and experimental deposition measurements.

### 8.4 Coupling deposition with pharmacokinetics and biological response

Deposition is only the first stage of local exposure. Regional pulmonary pharmacokinetic models can include dissolution, mucociliary clearance, epithelial permeation, tissue binding, metabolism, macrophage uptake, lymphatic transport, and systemic absorption, depending on the formulation and modeling objective (Borghardt et al., 2015). Physiologically based pharmacokinetic models can link lung compartments to systemic plasma and tissue exposure. For nanoparticles, additional state variables may be needed to distinguish intact carriers from released cargo and, where supported by quantitative data, describe cell-specific uptake or lymphatic transport.

Coupling physical deposition with organ-chip biology could provide a practical multiscale framework. The pulmonary model predicts the regional deposited dose under a given device, formulation, and inhalation maneuver. A chip reproducing key biological features of that region can then be exposed to an experimentally matched deposited dose and used to measure mucus transport, barrier responses, target engagement, inflammation, and toxicity. These data could inform biological parameter estimation or be used to train response surrogates. Such a coupled framework could compare formulations that have similar total deposition but different regional or cellular effects.

### 8.5 What qualifies as a pulmonary digital twin?

A patient-specific simulation is not automatically a digital twin. A clinically meaningful twin requires a defined physical or biological counterpart, longitudinal updating with patient-specific data, predictive capability, and a bidirectional information pathway through which model outputs can inform patient management; uncertainty should also be quantified for the intended use (Gonsard et al., 2024). A static CT-based deposition map is more accurately described as a personalized *in silico* model. This terminology matters because “digital twin” implies a level of dynamic integration, decision support, and validation that many current systems described as digital twins have not achieved.

A pulmonary twin for inhaled therapy might integrate airway imaging, lung function, inhaler technique, connected-inhaler or other device data, symptoms, exacerbation state, biomarkers, and treatment response. The twin could update estimates of regional deposition and pharmacological response, compare alternative devices or inhalation maneuvers, and quantify uncertainty. It might recommend a device, inhaler-training intervention, formulation, or dose, but only after prospective validation demonstrates that model-informed recommendations improve a predefined, clinically relevant outcome.

Biological updating remains challenging. Imaging and spirometry provide organ-level information but do not directly measure mucus rheology, epithelial state, or macrophage function. Patient-derived cells, sputum, exhaled biomarkers, or blood markers could provide partial biological inputs. A coupled physical–biological twin could therefore combine a deposition model with a patient-relevant organ-chip model or calibrated biological-response model. This is a plausible development direction, but it remains an emerging concept rather than a prospectively validated clinical platform.

### 8.6 Uncertainty, identifiability, and validation

A detailed model can produce precise-looking results even when parameters are structurally or practically non-identifiable. Airway boundary conditions, distal geometry, mucus distribution, particle hygroscopicity, and breathing behavior may each introduce uncertainty. Parameter identifiability and sensitivity should be evaluated, and outputs should include appropriate uncertainty intervals—such as credible intervals for Bayesian models—or clearly defined scenario ranges (Read et al., 2020). Model calibration should use data appropriate for parameter estimation, whereas validation should use data not involved in calibration or model selection whenever possible and should occur at levels relevant to the intended use, such as airflow, total deposition, regional deposition, pharmacokinetics, or biological response (Morrison et al., 2019).

Prospective validation is a critical step for models intended to support clinical decisions. A model may reproduce historical data but fail when the device, formulation, disease state, or target population changes. Validation datasets should adequately represent variation in anatomy, disease severity, sex, age, and inhalation behavior relevant to the intended use, with performance and calibration assessed in clinically important subgroups. Fairness is relevant because advanced imaging and connected-device data may be less accessible to underserved populations (Wilson et al., 2024). A twin that depends on data available only in specialist centers may widen rather than reduce treatment disparities. Sections 6–8 describe methods that address different stages of inhalable-biomaterial development, from formulation optimization to human-relevant validation and patient-specific deposition modeling. Table 3 compares their roles, required inputs, typical outputs, strengths, and practical limitations.

**TABLE 3:** Intelligence-enabled development and validation toolbox for smart inhalable biomaterials.

Tool/approach	Main purpose	Key inputs	Typical outputs	Strengths	Best use stage/limitations
Design of experiments	Efficient structured exploration of formulation space	Material composition; process variables; device conditions	Initial design map; main effects; interactions	Interpretable and experimentally practical	Best for early development; may miss complex nonlinear optima
Supervised learning	Predict structure-property-performance relationships	Material; aerosol; and biological descriptors	Predicted size; stability; aerosol metrics; uptake; toxicity	Captures multivariable relationships	Useful with quality datasets; vulnerable to data leakage and bias
Active learning/Bayesian optimization	Select most informative next experiments	Initial dataset plus uncertainty-aware surrogate model	Prioritized candidate formulations	Reduces experimental burden	Needs careful constraints and human oversight
Mechanistic aerosol modeling/CFD	Estimate regional deposition and airflow-dependent transport	Geometry; particle properties; inhalation waveform; device plume	Regional dose maps; hotspot analysis	Physics-based interpretability	Computationally intensive; depends on assumptions and boundary conditions
Reduced-order/surrogate models	Rapid approximation of deposition or response	High-fidelity simulations or curated experiments	Fast predictions with a defined applicability domain	Enables screening of many candidates	Requires uncertainty reporting and validation
Lung-on-chip validation	Human-relevant testing of deposited aerosol effects	Emitted formulation; deposited dose; airway cells; mucus; flow or strain	Barrier function; mucus transport; immune response; repeated-dose safety	Bridges static culture and *in vivo* testing	Lower throughput and higher complexity than simple cell culture
Patient-specific pulmonary modeling	Personalized estimation of delivery under real airway geometry	CT or imaging; lung function; inhalation behavior	Patient-level deposition estimates; device comparison	Supports personalization and decision-making	Requires validated inputs and careful uncertainty handling
Pulmonary digital twin	Longitudinally updated decision-support system	Imaging; physiology; inhaler-use data; biomarkers; treatment response	Updated predictions and model-informed recommendations	Most comprehensive closed-loop framework	Aspirational; requires continuous updating, validation, and governance

## 9 A closed-loop design–delivery–validation workflow

The methods summarized in Table 3 address different stages of inhalable-biomaterial development, but their value depends on whether findings at one stage inform decisions at the next. We therefore organize them into a development workflow that begins with the dominant delivery problem and links material design, aerosol characterization, human-relevant testing, and patient-specific deposition through iterative refinement.

### 9.1 Defining the clinical-engineering bottleneck

Development starts by identifying the dominant reason why an otherwise promising therapy may fail after inhalation, rather than by selecting a material first. Relevant bottlenecks may include inadequate small-airway deposition, trapping within mucus plugs, rapid luminal clearance, failure to reach the target macrophage population, premature release during nebulization, or repeated-dose epithelial toxicity. Defining the target cell, airway compartment, disease state, and intended device at this stage establishes the context in which material performance will be judged. The proposed material properties should then be linked mechanistically to the intended biological outcome.

### 9.2 Translating the bottleneck into measurable specifications

The identified bottleneck must next be expressed in measurable terms. Depending on the intended application, relevant specifications may include aerodynamic size, emitted dose, diffusion in disease-relevant mucus, off-target release under non-diseased conditions, trigger-activation threshold, intracellular release time, target-cell uptake, barrier recovery, or repeated-dose safety. Not every variable needs to be optimized at the outset; priority should be given to measurements that directly determine whether the dominant failure mode has been overcome.

### 9.3 Establishing the experimental and computational design space

The initial design space can include material composition, process variables, and device conditions, with physicochemical and mechanistic constraints used to remove combinations that are not experimentally or biologically plausible. Early experiments can establish particle formation, stability, aerosol performance, and basic biological compatibility. These results provide the first structured dataset for an interpretable surrogate model, after which active learning can be used to direct additional experiments toward uncertain or promising regions of the design space.

### 9.4 Evaluating the emitted formulation in human-relevant systems

Evaluation at this stage should focus on the formulation that actually emerges from the intended delivery device, rather than on the pre-aerosol material alone. Post-emission characterization and deposited-dose measurements can be coupled with air–liquid-interface cultures or lung-on-chip models to examine mucus transport, barrier function, target engagement, and toxicity under disease-relevant inflammatory and mechanical conditions. Once the platform is technically reproducible, incorporating multiple donors becomes important for determining whether performance is robust to biological variability. Poorly performing formulations are also informative because they help define the boundaries of the performance landscape.

### 9.5 Incorporating patient-specific deposition

Patient-specific or representative disease geometries provide a means to test whether candidate performance remains favorable when anatomical and inhalation heterogeneity are introduced. Regional dose estimates should incorporate model uncertainty when this uncertainty could affect candidate ranking. The relevant question is therefore not whether a model can generate a detailed deposition map, but whether accounting for realistic airway heterogeneity changes a formulation or device decision. A candidate that performs well in an idealized lung but provides insufficient exposure to obstructed small airways may consequently lose priority.

### 9.6 Iterative refinement and convergence

Results from aerosol testing, organ-chip experiments, and deposition modeling can be returned to the design stage to refine influential parameters and guide the next experiments. The design space progressively narrows as poorly performing regions are excluded and uncertainty around promising candidates decreases. A practical endpoint is reached when additional optimization produces little meaningful gain and the selected candidate satisfies predefined material, aerosol, biological, safety, and manufacturing requirements with uncertainty appropriate to its intended use. This iterative process can reduce unnecessary experimentation, but it does not replace applicable animal studies, formal toxicology, or clinical evaluation required for the final product.

### 9.7 Worked example of end-to-end closed-loop implementation

To illustrate how the proposed framework could operate in practice, we consider a hypothetical but evidence-anchored development scenario involving severe type 2-high asthma with persistent mucus plugging. This example is not intended to represent a clinically validated development pathway. Rather, it demonstrates how a disease-specific delivery bottleneck can be translated into quantitative engineering specifications, linked to material and device design, evaluated in human-relevant experimental systems, and iteratively refined using computational models.

The workflow would begin by defining persistent mucus plugging as the dominant clinical-engineering bottleneck rather than selecting a carrier *a priori*. Mucus plugs can persist within the same airway segments and are associated with airflow obstruction, ventilation defects, and type 2 inflammation (Tamura et al., 2022; Tran et al., 2022; Tanabe et al., 2025; Aegerter et al., 2026). Imaging could be used to characterize plug burden, airway narrowing, and the anatomical distribution of poorly ventilated regions, while patient-derived sputum or airway secretions could be characterized for solids content, rheology, pH, ionic conditions, and particle transport. These measurements would then be translated into predefined material-performance specifications, such as acceptable post-aerosol diffusion coefficients, mobile fraction, penetration depth, retention, and epithelial exposure.

A mucus-penetrating or sequentially transformable polymeric or lipid carrier could subsequently be developed, with particle size, surface shielding, coating density, cargo loading, and post-deposition transformation treated as design variables rather than fixed attributes (Schneider et al., 2017; Kim et al., 2022). Device-related variables should be incorporated into the same design space. For example, dry-powder, soft-mist, or nebulized delivery could be compared using clinically relevant inhalation waveforms, emitted dose, aerodynamic particle-size distribution, and post-device carrier integrity. An initial structured dataset linking formulation and device variables to outputs such as MMAD, FPF, mucus transport, epithelial uptake or target engagement, potency, and toxicity could then support data-driven candidate ranking. Where prospective iteration is feasible, active learning or Bayesian optimization could prioritize subsequent candidates according to multiple objectives rather than optimizing a single aerosol or nanoparticle metric.

The selected formulations would then be evaluated after passage through the intended inhalation device using a mucociliary airway-chip or related air–liquid-interface system. Quantified deposited dose, mucus transport, epithelial target engagement, barrier function, ciliary behavior, inflammatory responses, and repeated-exposure effects could be measured under type 2-like or patient-derived conditions (Sengupta et al., 2023; Liu et al., 2025). In parallel, CT-based patient-specific or representative-disease computational fluid dynamics could estimate regional deposition under alternative particle-size distributions, devices, and inhalation maneuvers (Sadafi et al., 2024a; Sadafi et al., 2024b). This step could reveal failure modes that would not be apparent from conventional formulation testing alone. For example, a candidate with a high FPF could still be deprioritized if regional modeling predicted insufficient exposure in mucus-obstructed target airways, whereas a formulation with favorable regional deposition could fail if the deposited carrier remained trapped within disease-relevant mucus or produced inadequate epithelial target engagement.

The aerosol, airway-chip, and deposition-model outputs would then be returned to the design model to update candidate ranking and determine the next experimental round. Depending on the identified failure mode, the subsequent iteration might modify surface shielding, particle or aerodynamic size, transformation kinetics, cargo dose, device selection, or inhalation conditions. Iteration would continue until additional optimization produced little meaningful improvement and a candidate satisfied predefined aerosol, mucus-transport, biological, safety, and delivery requirements within an acceptable uncertainty range. This example illustrates the central principle of the proposed closed-loop framework: material composition, aerosol behavior, biological performance, and patient-specific regional exposure are not optimized as independent endpoints, but are connected through sequential decisions in which the results of one stage directly inform what is tested next.

## 10 Translational, manufacturing, regulatory, and health-system considerations

### 10.1 Chemistry, manufacturing, and controls

Complex inhaled biomaterials may have multiple potential critical quality attributes, depending on the material platform and intended product profile, including material identity, component molecular weight where relevant, composition, size distribution, morphology, surface chemistry, ligand density, cargo loading or encapsulation efficiency, release profile, sterility, bacterial endotoxin level, residual solvents, moisture, solid-state properties, aerosol performance, and biological potency (Taha et al., 2020). Responsive function itself may be a critical quality attribute when the trigger threshold, response magnitude, or response kinetics affect product safety or efficacy. A batch can meet size and loading specifications yet fail to respond at the intended trigger threshold.

Scalable manufacturing should be considered early. Laboratory sonication, small-batch solvent evaporation, or manual membrane extrusion may not translate directly to scaled or GMP-compliant manufacturing. Mixing, drying, sterilization, and filling can change material structure and performance. Sterilizing-grade filtration may be unsuitable for particulate formulations whose size distribution, aggregation tendency, or membrane interactions compromise filterability or product recovery, whereas terminal sterilization may damage polymers, lipids, proteins, nucleic acids, or responsive functionalities (Troiano et al., 2016). For biologically sensitive carriers, a practical industrial strategy is therefore to build sterility into the manufacturing process rather than rely on sterilization of the final product. Compatible aqueous components can be sterilizing-filtered before particle assembly, followed by formulation, purification, buffer exchange, and transfer within validated closed or single-use processing systems. Where the final particle size and formulation properties permit, 0.22-μm sterilizing-grade filtration of the assembled product can remain feasible, but filter material, capacity, fouling, product recovery, and post-filtration critical quality attributes should be validated for the specific formulation (Wu et al., 2025). When final filtration is not technically compatible, aseptic assembly from appropriately controlled inputs followed by aseptic filling in isolator- or RABS-based systems provides a more realistic route for nucleic-acid- or protein-containing responsive carriers. Industrial implementation additionally requires control of raw-material bioburden and endotoxin, minimized open manipulations, validated hold times and aseptic process simulations, environmental and process monitoring, and maintenance of container–closure integrity. Such strategies shift sterility assurance from a single terminal step to a controlled end-to-end manufacturing process while preserving carrier structure and biological potency. Process analytical technology and multivariate control can support consistency, but only if relationships between process variables and product performance are established.

### 10.2 Storage, packaging, and device integration

Storage conditions can promote oxidation, hydrolysis, aggregation, changes in crystallinity or moisture content, and loss of biological activity. Dry powders may improve stability but can remain sensitive to humidity. Liquid nanoparticle formulations may require refrigeration and protection from light or oxygen. Packaging materials may adsorb drug or formulation components or release leachables. Device reservoirs, valves, meshes, and tubing can interact with the formulation.

Combination-product development requires coordinated control of the formulation and device. Changing the device can alter emitted dose and regional deposition even when the formulation composition is unchanged. Human-factors testing is particularly important when the intended users include older patients or patients with severe COPD, or when use during acute exacerbations is anticipated. Connected features can support training and monitoring, but cybersecurity, interoperability, and data governance become additional development and risk-management requirements.

### 10.3 Safety and immunological risk

Pulmonary safety can be compromised by persistent particles, endotoxin contamination, complement activation, and other pro-inflammatory impurities. Safety studies should distinguish toxicity attributable to the carrier from that attributable to the cargo or contaminants. Particle overload can impair alveolar macrophage clearance and function. Cationic components may disrupt cell membranes, depending on surface charge density and dose. Degradation products may alter local pH or induce inflammation. Biologically derived materials may contain potentially immunogenic proteins or nucleic acids. Repeated administration may elicit adaptive immune responses against proteins, polymers, or surface ligands.

Host defense is a particular concern. Depending on the target and degree of inhibition, suppressing neutrophil function, ROS production, protease activity, or inflammatory signaling may reduce tissue injury but may also compromise antimicrobial defense. Studies should therefore evaluate antimicrobial competence or responses to infection when the intended mechanism intersects with host defense. Persistent bacterial colonization occurs in some COPD phenotypes, and patients may also receive corticosteroids or biologics that modify immune responses.

### 10.4 Preclinical evidence hierarchy

A robust preclinical-to-clinical evidence package can progress, as appropriate to the intended use, from material characterization to aerosol testing, evaluation in disease-relevant mucus, differentiated air–liquid-interface cultures, dynamic lung-on-chip systems, appropriately designed animal inhalation studies, toxicology, and ultimately clinical evaluation. Intratracheal instillation can demonstrate biological plausibility but should not be considered equivalent to aerosol inhalation. Animal models provide whole-organ and immune-system interactions but differ from humans in airway branching, mucus properties, breathing patterns, and immune biology. Human-relevant chips and computational models are complementary tools that can help address, but not fully eliminate, these species gaps (Cidem et al., 2020).

Validation should be mechanism-specific. A mucus-penetrating claim requires demonstration of transport in disease-relevant mucus and improved tissue access after aerosolization. A targeting claim requires comparison with an otherwise matched non-targeted control and evidence of target-dependent interaction or efficacy. A responsive claim requires characterization of activation thresholds and response kinetics under relevant conditions. An intelligence-enabled claim requires validation using independent data and, when improved experimental efficiency or candidate selection is claimed, evidence that the model provided that benefit (Schneider et al., 2017).

### 10.5 Regulatory evidence for AI-enabled development

When machine learning is used solely as an internal development tool to select a formulation and its outputs are not relied upon for regulatory decision-making, the primary regulatory focus generally remains the quality and performance of the resulting product, although model documentation supports development traceability. If model outputs are used to support regulatory decisions regarding product quality, safety, or effectiveness, the model should be assessed according to its context of use and associated risk. When an algorithm becomes part of the marketed product—for example, by recommending a dose or device—it may fall within medical-device software requirements or form part of a combination product, depending on its functionality and the applicable jurisdiction; its intended use, update strategy, performance monitoring, human oversight, and management of performance drift should then be specified.

Digital-twin claims require particularly careful evidence. The model should have a predefined context of use and decision, well-characterized and validated input pipelines, quantified uncertainty, and prospective evaluation when intended to guide clinical decisions. A model that merely visualizes airflow or provides a one-time patient-specific simulation should not be presented as a clinical digital twin. Transparent reporting of model maturity helps distinguish decision-oriented, dynamically updated patient-specific models from exploratory simulations and reduces overclaiming (Sel et al., 2025).

### 10.6 Health-system implementation and equity

Health-system implementation should be considered alongside technological development because the value of intelligence-enabled inhaled therapies ultimately depends on whether they can be integrated into real-world clinical workflows. Intelligence-enabled development could shorten formulation-development cycles, while patient-specific modeling and connected-inhaler data could support device selection, improve inhaler training, identify patients or situations at risk of underdelivery, and enable remote monitoring. Realizing these potential benefits will require integration with clinical workflows, reimbursement pathways, technical support, and appropriate data infrastructure.

Equity should be considered throughout development and implementation. Patient-specific modeling may require access to high-quality imaging, lung-function testing, smartphones, connected devices, or specialist care. Populations with limited access to these resources may be underrepresented in training datasets and may be less able to benefit from the resulting system. Robust operating modes that require fewer or less complex data inputs, transparent reporting of performance across relevant population subgroups, and alternatives that do not depend on advanced infrastructure should therefore be planned. Material innovation should not depend on a digital layer that reduces accessibility or widens existing disparities.

## 11 Priority research directions

Progress in this field will depend in part on more comparable experimental systems and reference materials. Standardized mucus surrogates should be complemented by well-characterized human samples representing different asthma and COPD phenotypes (Lock et al., 2018; Chisholm et al., 2019). Benchmark particles with defined mucus-penetration and adhesion profiles could support interlaboratory comparisons, while aerosol exposure studies should report both deposited dose and overall mass recovery (Ding et al., 2020).

Comparable experimental systems will have limited value, however, unless the resulting data can also be reused across studies. Prospective multi-laboratory datasets containing harmonized metadata, negative results, raw aerosol distributions, and relevant biological context would provide a stronger basis for generalizable model development than isolated reports of high-performing models (Jiang et al., 2022; Narayanan et al., 2021). External benchmarking using data from independent laboratories and previously unseen material classes would provide a more meaningful test of model generalizability.

Responsive-material studies also need to move beyond qualitative demonstrations of stimulus sensitivity. Measurements in human airway fluids should define physiologically and pathologically relevant ranges of oxidants, reducing conditions, enzyme activities, pH, and mucus properties (Wilson et al., 2010; Pniewska and Pawliczak, 2013; McKelvey et al., 2021). Response thresholds can then be evaluated across relevant patient subgroups and disease states. Such measurements are necessary to establish whether a responsive mechanism is likely to operate under clinically relevant conditions or whether a simpler sustained-release formulation would be sufficient.

For biological validation, greater model complexity is useful only when it answers a defined translational question without compromising reproducibility. Lung-on-chip platforms may therefore need to integrate aerosol dosimetry, mucus transport, immune cells, and repeated exposure according to the intended application. Higher-throughput formats and automation could make these systems more practical for formulation optimization. Patient-derived chip studies should include sufficient independent donors to characterize response heterogeneity, with statistical designs that account for technical chips or repeated measurements nested within donors.

A similar principle applies to patient-specific pulmonary modeling: deposition predictions are most informative when they are connected to a biological or therapeutic consequence. Regional dose estimates can inform organ-chip exposures, pharmacokinetic models, and device selection rather than ending with airflow or deposition visualization alone. Reduced-order models should quantify predictive uncertainty and undergo prospective validation for their intended use. The term digital twin should remain restricted to longitudinally updated, uncertainty-aware systems linked to a defined decision rather than one-time patient-specific simulations.

Across these directions, increasing technical sophistication should not become an objective in itself. A carrier that combines mucus penetration, responsive release, targeting, imaging, and catalytic activity may be scientifically impressive but difficult to characterize, manufacture, and validate. From a translational perspective, the preferred design is the least complex architecture that reliably overcomes the dominant delivery barrier, survives aerosolization, provides a measurable biological advantage, and can be manufactured reproducibly at scale.

## 12 Conclusion

Smart inhalable biomaterials may improve therapy for asthma and COPD, but their translational value depends less on adding functions than on linking material design to the actual barriers that determine pulmonary exposure. Material chemistry, aerosol behavior, device performance, and patient-specific inhalation conditions therefore need to be considered as parts of the same delivery problem, while biological effects should be evaluated at a known deposited dose in human-relevant systems. Computational and experimental tools can support this process, but only when their outputs are used to inform subsequent design decisions rather than treated as separate demonstrations of technical capability. Machine learning and mechanistic modeling can help prioritize experiments and formulations, lung-on-chip systems can test post-aerosol biological responses under more realistic conditions, and patient-specific pulmonary models can determine whether anatomical and inhalation heterogeneity materially alter regional exposure. The practical goal is not to maximize the complexity of the material or the model, but to identify the simplest design that reliably overcomes the dominant delivery barrier and retains its function after aerosolization. A pulmonary digital twin remains a longer-term objective that will require longitudinal updating, uncertainty quantification, prospective validation, and linkage to a defined clinical decision. In the near term, the more attainable objective is to replace fragmented empirical optimization with an integrated development pathway in which material performance, aerosol delivery, biological response, and patient-specific exposure are evaluated together.
